# Review of the Applications of Biomedical Compositions Containing Hydroxyapatite and Collagen Modified by Bioactive Components

**DOI:** 10.3390/ma14092096

**Published:** 2021-04-21

**Authors:** Agnieszka Sobczak-Kupiec, Anna Drabczyk, Wioletta Florkiewicz, Magdalena Głąb, Sonia Kudłacik-Kramarczyk, Dagmara Słota, Agnieszka Tomala, Bożena Tyliszczak

**Affiliations:** Department of Materials Science, Faculty of Materials Engineering and Physics, Cracow University of Technology, 37 Jana Pawła II Av., 31-864 Krakow, Poland; agnieszka.sobczak-kupiec@pk.edu.pl (A.S.-K.); anna.drabczyk2@pk.edu.pl (A.D.); wioletta.florkiewicz@pk.edu.pl (W.F.); magdalena.glab@doktorant.pk.edu.pl (M.G.); sonia.kudlacik-kramarczyk@pk.edu.pl (S.K.-K.); dagmara.slota@doktorant.pk.edu.pl (D.S.); aatomala@gmail.com (A.T.)

**Keywords:** collagen, hydroxyapatite, drugs, bioactive components, metals, nanoparticles, regenerative medicine, tissue engineering

## Abstract

Regenerative medicine is becoming a rapidly evolving technique in today’s biomedical progress scenario. Scientists around the world suggest the use of naturally synthesized biomaterials to repair and heal damaged cells. Hydroxyapatite (HAp) has the potential to replace drugs in biomedical engineering and regenerative drugs. HAp is easily biodegradable, biocompatible, and correlated with macromolecules, which facilitates their incorporation into inorganic materials. This review article provides extensive knowledge on HAp and collagen-containing compositions modified with drugs, bioactive components, metals, and selected nanoparticles. Such compositions consisting of HAp and collagen modified with various additives are used in a variety of biomedical applications such as bone tissue engineering, vascular transplantation, cartilage, and other implantable biomedical devices.

## 1. Introduction

Tissue engineering is a modern scientific discipline concerning chemical, biological, and engineering principles that tries to use various methods to regenerate tissues [1,2]. It applies natural science and engineering principles and their innovations to such damaged tissues through the development of biological substitutes or through tissue reconstruction [3,4]. To revitalize, maintain, or enhance tissue function, tissue engineering helps to understand the structure and function of normal and pathological mammalian tissues [1,2,5,6]. The aim of tissue engineering is to develop new functional tissues and regenerate tissues in vitro or in vivo to treat diseases when surgery is necessary [7]. For many other disease states, tissue engineering remains a thriving area of research with potential new treatments [8]. It enables the regeneration of almost every tissue and organ in the human body [6]. General tissue-engineering strategies can be divided into three groups: (i) implantation of isolated cells into the body or cell replacement; (ii) delivery of tissue-inducing substances such as growth factor, which refers to proteins or polypeptides that can promote tissue growth; and (iii) placing cells in or on different matrices [6]. Tissue engineering is broadly divided into two types: (a) soft tissue engineering, which deals with skin, blood vessels, tendons/ligaments, cardiac lobe, nerves, and skeletal muscles; and (b) hard-tissue engineering, dealing with bones [9]. Bone-tissue engineering aims to induce ideal bone healing by using hybrid matrixes of osteoconductive and biodegradable biomaterials and osteoinductive growth factors [10,11]. In the area of bone-tissue engineering, the biomimetic scaffolds are designed to receive the artificial bone matrix and to support the adhesion of cells, followed by the process of new tissue formation [12]. Therefore, it is assumed that the scaffold is to imitate as much as possible the structure and the composition of natural tissues [13]. In recent years, scaffolds based on natural polymers, synthetic ones, or their adequate combinations are of great interest [14]. Of particular interest is a combination of synthetic polymers, which provide adequate mechanical strength and processability, with biopolymers, which ensure the cells have an appropriate environment for proliferation and the induction of tissue growth [15].

## 2. Hydroxyapatite

Hydroxyapatite (HAp) is a biologically active calcium phosphate ceramic with high ability to promote bone growth; however, its mechanical properties, such as low fracture toughness, poor tensile strength, and weak wear resistance, make it insufficient as a load-carrying material [16]. The literature refers to hydroxyapatite as HAP, HAp, HA, or OHAp [17,18,19]. Apatites are widespread minerals occurring in all types of rocks, mainly in Switzerland, Spain, Canada, Brazil, Australia, and Poland [18]. Biological apatites are found mainly in bones and teeth of vertebrates. They are also present in all pathologically calcified tissues, such as salivary, cerebral, kidney, bile, ureteral, and tartar stones. The apatite of which bones are made is referred to in the literature as “bone hydroxyapatite” [20,21,22]. The size of hydroxyapatite crystals in human bones depends on the age. Three characteristic ranges of average crystallite size can be identified: 188–215 nm—the childhood period under 6 years; 232–252 nm—the adolescent period of 6–19 years; and—252–283 nm—maturity [22,23]. Bone apatites are mainly acicular or lamellar crystals [24].

**Figure 1:**
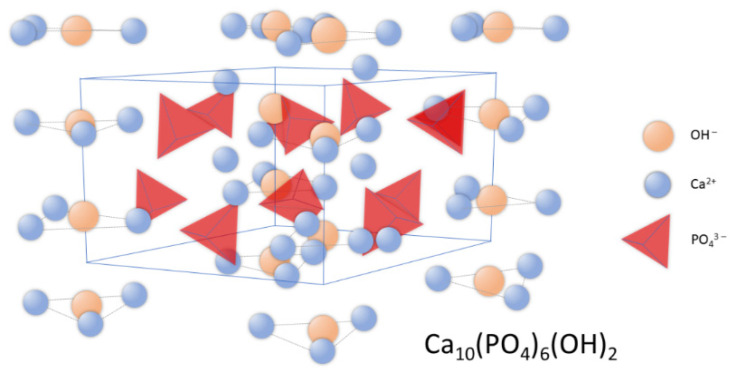
Schematic representation of hydroxyapatite’s crystal structure.

Synthetic apatites are a group of compounds that includes both stoichiometric hydroxyapatite, s-HAp, with a molar ratio of calcium to phosphorus equal to 1.667; as well as hydroxyapatite deviating from stoichiometry (ns-HAp). ns-HAp shows a very wide range of nonstoichiometry, as it can contain mainly water or HPO_4_^2−^, H_2_PO_4−_ ions, while OH^−^ can be replaced by O^2−^ [25,26,27,28]. Stoichiometric hydroxyapatite has a monoclinic structure, while HAp of mineralogical and biological origin has a hexagonal structure [29]. The unit cell parameters for the hexagonal system are: a = 9.41 Å, c = 6.88 Å, and the unit cell volume is 527.59 Å³ [18]; while for the monoclinic system, they are: a = 9.4215 Å, b = 2a, c = 6, 8815 Å [30]. Figure 1 shows the distribution of atoms in the hydroxyapatite crystal lattice.

In the unit cell, two crystallographically independent atoms of calcium, Ca (I) and Ca (II), can be observed. Ca (II) atoms form triangles that are located perpendicular to the c axis and mutually shifted by 60° to each other, while Ca (I) atoms are octahedrally surrounded by six oxygen atoms [31]. The structure of hydroxyapatite adopts a variety of isomorphic substitutions in both the cationic and anionic subnetworks without destroying the unit cell structure. The criteria determining the possibility of ion exchange are the similarity in dimensions and charges of the substituting and substituted ions [22,32,33]. Foreign elements are substituted into the structure of hydroxyapatite in an undefined amount, depending on the conditions of its formation [34].

In the structure of hydroxyapatite, the anions of PO_4_^3−^ can to some extent be exchanged for carbonate groups, and this is the so-called Type B carbonate hydroxyapatite, unlike type A, in which CO_3_^2−^ anions replace the hydroxyl groups [34,35]. Type A carbonate hydroxyapatite is obtained by high-temperature treatment >1000 °C. In naturally derived hydroxyapatite, carbonate anions can replace both the phosphate groups and the hydroxyl groups. Carbonate anions in biological hydroxyapatite are also adsorbed on its surface. Substitutions in the anionic subnetwork with other anions such as chlorine (0.13 wt %) or fluorine (0.03 wt %) are also possible. Calcium ions can be exchanged for magnesium (about 0.7% by weight); sodium (about 0.9% by weight); potassium (0.03%); and a number of trace elements (Sr, Pb, Zn, Cu, Fe) [36]. The presence of these elements affects the activity of enzymes related to the operation of bone cells. The incorporation of Mg^2+^ and CO_3_^2−^ ions causes a reduction in crystal size and an increase in solubility [37,38]. The effect of low crystallinity is high reactivity of bone apatites, which is reflected in bone-resorption processes. Foreign elements are incorporated in substitutions in amounts depending on the conditions of the formation of this structure; their presence also affects the stoichiometry (increasing the Ca/P molar ratio), crystallinity, and thermal and chemical stability of the compound [39].

There are molecular modeling methods that allow us to define the Hap structure. Bystrova et al. presented the first basic modeling and calculations for hydroxyapatite (HAP) nanostructures, as well as native, surface modified, charged, and with various defects (H and OH gaps, H internode) based on the first basic modeling [40].

There are many methods of obtaining hydroxyapatite powders, i.e., wet, dry, fluxing and sol-gel [25]. The mechanochemical method of obtaining hydroxyapatite is also known, but it has not found wide application [41]. Hydroxyapatite can also be obtained from natural materials such as corals, animal bones, and even fish bones [42,43,44,45]. The most widely used methods on an industrial and laboratory scale are wet methods. Particular methods allow the obtaining of materials with appropriate morphology, crystal structure, and Ca/P molar ratio, as well the incorporation of foreign cations or anions into the structure of hydroxyapatite [46,47].

Hydroxyapatite, due to its structural similarity to the mineral parts of natural bone, has been widely used in medicine and dentistry. The physicochemical and biological properties of hydroxyapatite make it possible to classify it as a bioactive material [48,49,50,51]. HAp has been applied in orthopedics, dentistry, maxillofacial surgery, ophthalmology, laryngology, and traumatology [25,32,52]. In medical practice, hydroxyapatite bioceramics are used in the forms of powder, granules, solid material, porous material, composite component, or a layer on various types of substrates [53].

## 3. Collagen

Due to its properties, collagen accounts for approximately 30% of all proteins found in vertebrate organisms. It is therefore a key and ubiquitous component of the extracellular matrix, providing the tensile strength required to meet the high biomechanical requirements of human and animal tissues.

Collagen, as the most abundant protein in the human body, has long been known as a natural material for a variety of biomedical applications, including implants and drug delivery [54,55]. An important source of type I collagen is animal skins. Physically, collagen forms a rodlike triple helix that self-assembles to form a cove D-periodic filament matrix. The fibers are cross-linked to provide mechanical extracellular matrix strength, integrity, and distribution filament diameters. The degree of cross-linking strongly affects the tensile strength and elasticity of tissues. Embossed collagen molecules or fibers can form hydrogels, membranes, or sponges, which can be used as hemostatic inserts, wound dressings, grafts, and scaffolds for surgery and tissue engineering [56,57,58,59].

**Figure 2:**
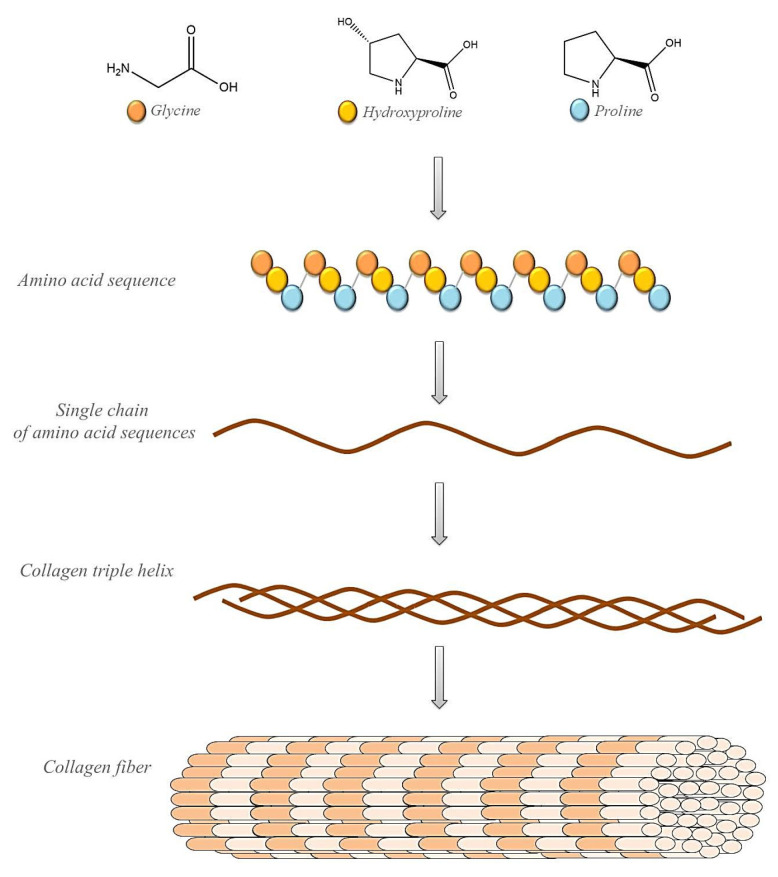
Schematic representation of collagen’s structure.

Collagen (Figure 2) is used as the main ingredient in many drug-delivery systems and biomaterials such as ointments and dressings. Its basic physical and structural properties, along with low immunogenicity and natural turnover, are the key to its biocompatibility and efficacy. The collagen triple helix can interact with a large number of molecules that trigger biological events. They regulate the interactions of collagen with receptors on the cell surface in many cellular processes. Collagen can also interact with enzymes involved in its biosynthesis and degradation. In recent years, many interactions between collagen and other molecules have been described. These studies determined the responsible sequences of collagen bonds and high-resolution structures of triple-helical peptides associated with their natural binding partners. Intelligent control of the biological interactions of collagen in a material context will increase the effectiveness of collagen-based drug delivery [56,57,58].

Currently, 26 genetically different types of collagen have been described. Taking into account the supramolecular structure and organization, they can be divided into fibril-forming collagens, fibril-bound collagens (FACIT), collagens formed by NETs, anchoring and transmembrane fibril collagens, basement-membrane collagens, and others with unique features (see Table 1).

Different types of collagen are characterized by high complexity and diversity in their structure, i.e., variants of their connection, the presence of additional ones, and nonhelical domains, as well as their assembly and function. The most numerous and widespread family of collagens containing approximately 90% of total collagen is represented by collagen-forming fibrils. Types I and V collagen fibrils contribute to the structure of bone spine, while type II and XI collagens mainly contribute to the filamentous matrix articular cartilage. Their torsional stability and extensibility strength lead to their stability and integrity tissues. Type IV collagens, with higher flexible triple helix combined into a mesh, are limited to foundation membranes. In contrast, the highly cross-linked disulfide is of the type VI collagen-forming microfiber. We also distinguish collagens associated with fibrils with intermittent triple helices (FACIT). This type of collagen includes collagens IX, XII, and XIV, which play a role in regulating the diameter of collagen fibers. Hexagonal lattices form collagens of types VIII and X, while collagens XIII and XVII even include cell membranes [60].

## 4. Compositions of Collagen/HAp

Tissue engineering and regenerative medicine are rapidly developing fields of science that enable the design of substitutes. In materials science, hydroxyapatite and collagen are known factors that improve bone regeneration. Compositions that contain hydroxyapatite ceramics and collagen have unique properties, such as biocompatibility, biodegradability, and mechanical strength. Such compositions should be biodegradable, nontoxic, and nonimmunogenic, and should show similar mechanical strength to the tissues they replace. The use of these two materials together shows a synergistic osteoconductive effect. The best results are obtained by using collagen–hydroxyapatite compositions modified with other active substances [61,62].

Various techniques have been used to produce collagen/HAp compositions applicable in tissue engineering. In recent times, the most popular methods include gel casting, compaction, computer-aided rapid prototyping (RP), and 3D printing. The possibility of producing materials with controlled mechanical properties and specific biological behavior is provided by collagen/HAp composition. The use of collagen resorbable matrices makes it possible to obtain multifunctional implants in which, after fulfilling the biomechanical function (tissue fixation) after the sorption process, the HAp phase can act as a scaffold for the growth of osteogenic cells. When designing a tissue-engineered scaffold, it must be remembered that its shape must conform to the damaged tissue that is to be replaced. The scaffold must also have appropriate structural and functional properties. Currently, much research is being conducted on bioactive scaffolds modified with growth factors that are able to accelerate cell multiplication and support tissue regeneration [63,64,65,66,67].

Due to their structure, collagen/HAp compositions have different physicochemical and biological properties. The finely fibrous collagen/HAp compositions are a good medium for in vitro cultivation of osteoblasts. In contrast, porous collagen/HAp composites provide a good substrate for proliferating and differentiating osteogenic bone marrow cells [61,68,69,70].

## 5. Conjugates for Drug Delivery

Drug-delivery systems are currently defined as systems or technology for achieving optimal therapeutic effects of drugs by precisely controlling their movements in the body. To optimize the operation evaluation and analysis, it is essential for such delivery systems to determine the drug-distribution profile. Currently, drug-delivery systems are designed based on the relationship between physicochemical properties and distribution profile [71,72]. Conjugates are complexes that contain drug carriers with which the drug is usually bound by a covalent bond. It is also possible to use proteins, peptides, or DNA as an active substance instead of a drug. Natural or synthetic polymers are used as macromolecules, but proteins and antibodies are also found. Binding of a drug to its carrier is often accompanied by an alteration of its distribution, which is beneficial when its increased accumulation in the target tissue is desirable. Biodistribution of a bound drug depends on the properties of the carrier [73,74,75].

### 5.1. Conjugates of Collagen/HAp/Drugs

Currently, much research is focused on drug-delivery systems. In the case of bone pathologies such as osteoporosis, osteomyelitis, and osteosarcoma, drug-delivery systems are sought to enable bone regeneration. Natural collagen and HAp-derived biomaterials play an important role in drug delivery and slow and long-term release. Drugs introduced into this type of biomaterial have different tasks. Antibiotics such as vancomycin, tetracycline, and gentamicin are incorporated into biomaterials to inhibit the growth of bacterial strains that have caused severe wound infections and to support tissue regeneration. Collagen/HAp/drug conjugates show high accumulation and retention at bone sites, and are effective for timely drug release. It is important to enclose the therapeutic compounds in the HAp/collagen compositions and introduce the drug directly into the bone [76,77,78,79,80,81,82,83,84,85].

#### 5.1.1. Conjugates of Collagen/HAp/Statins: Preparation and Application

**Figure 3:**
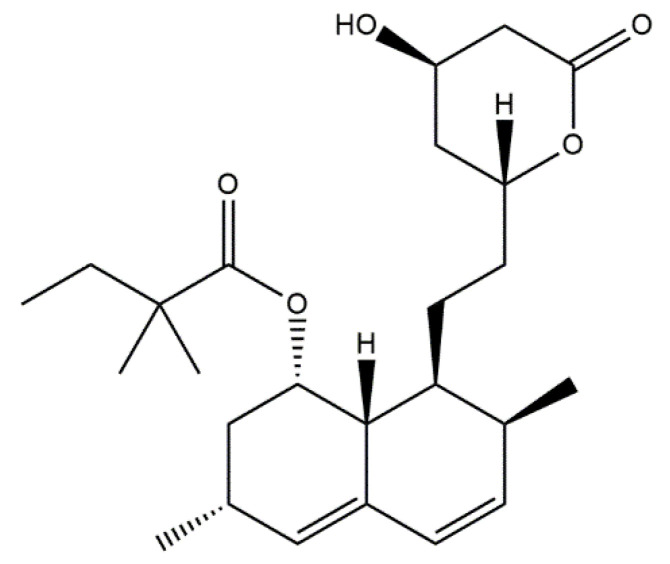
Schematic representation of simvastatin’s chemical structure.

Statins are a group of multifunctional organic chemicals used in medicine due to their broad therapeutic effects. It has been shown that selected statins positively influence the processes of bone formation and resorption; however, the mechanism of their action has not yet been thoroughly investigated [86]. A particularly interesting statin in the aspect of bone-system regeneration is simvastatin. It leads to the modulation of bone regeneration processes at the molecular and cellular level by inducing a pleiotropic effect [87]. In addition, it activates osteoblasts by increasing the expression of BMP-2 and inhibits osteoclasts, being bone cystic cells [88], and increases the secretion of vascular endothelial growth factor (VEGF) by stimulating neovascularization [89]. In 2013, Gao et al. [90] presented a combination of the described statin with hydroxyapatite. The aim of the study was to investigate the carrier properties of hydroxyapatite fibers by measuring the release of simvastatin (Figure 3) from these materials, and to evaluate the bone regeneration caused by the different mixtures of simvastatin/hydroxyapatite.

**Figure 4:**
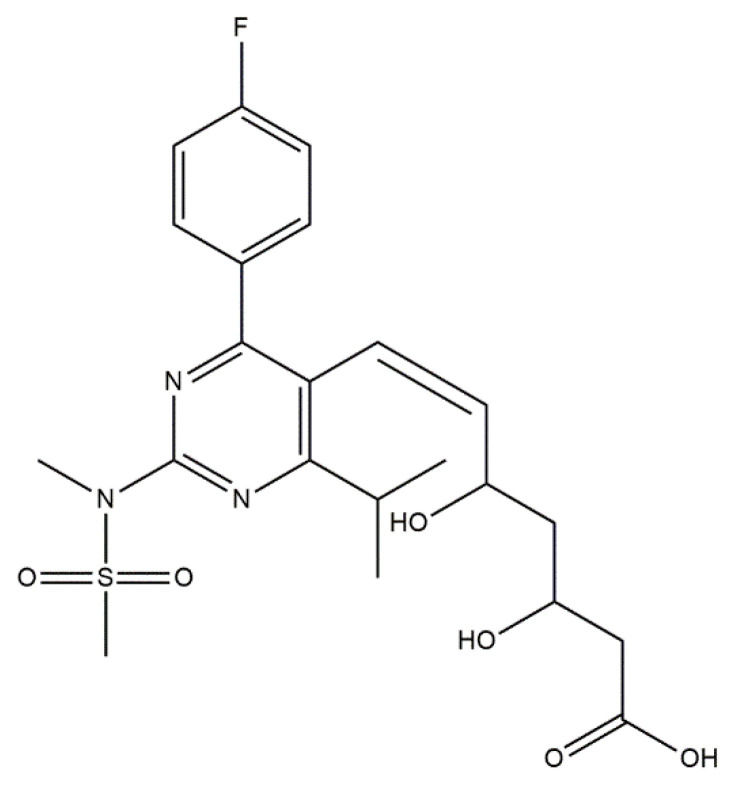
Schematic representation of the chemical structure of rosuvastatin (RVS).

Based on the in vivo study, it was confirmed that HAp can be used as a carrier for statin, and the optimal dose of the drug in the rabbit chamber model was determined. A new, three-component hydroxyapatite/collagen/simvastatin combination scaffold was first proposed in 2017 by Sun et al. [91] and others to stimulate angiogenesis and osteogenesis. In the study, the drug was closed in the microspheres of hydroxyapatite, and the body’s response to the combination of HAp/simvastatin against the three-component system with collagen was compared. The results were based on in vivo experiments on rats calvarias defects confirming that the proposed novel combination is promising in bone-tissue regeneration. A similar arrangement was proposed by enclosing simvastatin in mesoporous hydroxyapatite and suspending such obtained carrier in collagen matrix. This system allowed for the homogenous incorporation of water-insoluble simvastatin into the hydrophilic matrix, which resulted in a sustained release profile of the entrapped statin. As a result, the huge potential of designed scaffolds in repairing bone defects by simultaneously increasing osteogenesis and angiogenesis was confirmed [92]. Another interesting variation is the combination of simvastatin with deproteinized bovine bone with hydroxyapatite/β-tricalcium phosphate biphasic ceramics and with a collagen sponge. Such combination was analyzed to evaluate the local effect of the drug on bone repair in critical size defects in rat calvaria. Histometric analysis showed that the use of ceramics containing selected statin increased the formation of bone tissue in defects compared to the use of unmodified ceramic materials. Thus, the concept is promising and possesses a large application potential. Furthermore, the use of biomaterial used on the market affects the costs of the whole composite [93]. Experimentally, Monjo et al. [94] decided to investigate the effect of another statin drug, i.e., synthetic rosuvastatin (RVS) (Figure 4), on bone formation. Compared to the aforementioned lipophilic simvastatin, RVS is a hydrophilic statin, mostly used to prevent cardiovascular diseases at high risk, as well as to treat abnormal lipid metabolism [95]. However, the authors first demonstrated the ability of RVS to promote the expression of bone morphogenetic protein BMP-2 and osteoblast differentiation. The objective of the work they presented was to evaluate the potential of an absorbable collagen sponge as a carrier for RVS to enhance bone formation in critical-size cortical bone defects adjacent to titanium implants. The biomaterial was placed in an area of previously removed bone marrow in the tibial bone of New Zealand White rabbits. The results obtained confirmed that RSV, applied locally in the bone, had the potential to stimulate new bone formation.

#### 5.1.2. Conjugates of Collagen/HAp/Paclitaxel: Preparation and Application

**Figure 5:**
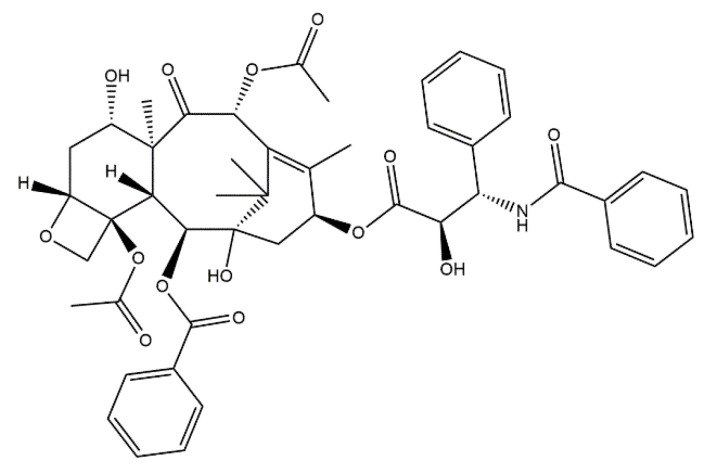
Schematic representation of paclitaxel’s chemical structure.

Paclitaxel (Figure 5) is a well-known anticancer drug that is considered as one of the most effective active substances applied in the treatment of the mentioned disease available on the drug market [96]. It is a key chemotherapeutic agent in the therapy against malignant tumors including breast ovarian, lung or Kaposi sarcoma. Paclitaxel has been approved for the use by the U.S. Food and Drug Administration (FDA) [97]. Furthermore, this drug is also used in the treatment of the stomach, esophagus, uterus, and prostate [98]. Paclitaxel is considered as a means of causing the death of cells. In recent years, numerous investigations on the systems delivering this anticancer drug aimed to prevent the side effects, while simultaneously, intensity of its anticancer activity was investigated [99]. An interesting solution is the combination of paclitaxel with calcium phosphate cements [100], TiO_2_ nanoparticles [101], or hydroxyapatite [102].

In many cases of cancer diseases, bone metastases constitute a meaningful problem. The removal of bone metastases significantly weakens the bone, and the resulting defects must be filled. Materials applied for such filling provide adequate mechanical strength of bones together with the possibility of local delivery of anticancer drugs [103]. In the case of bone defects caused by cancer diseases, the use of the materials containing both hydroxyapatite to support bone regeneration and paclitaxel as an anticancer drug constitutes an interesting solution with a great application potential. For example, Venkatasubbu et al. [104] developed a system consisting of poly(ethylene glycol) modified with hydroxyapatite nanoparticles functionalized with folic acid. Subsequently, paclitaxel has been introduced into such developed materials. Both surface modification and functionalization was confirmed by FT-IR spectroscopy, thermogravimetric analysis, and UV spectroscopy. Furthermore, the release profile of this anticancer drug has also been determined. The results obtained indicated that at the beginning of the study, the drug release was rapid, and thus the subsequent release of paclitaxel was stable and steady. The investigations on the release profile indicated that 100% release of the anticancer drug occurs within 50 h. On the other hand, Srivastav et al. [105] focused on the comparison of different forms of hydroxyapatite as carriers of anticancer drugs. Paclitaxel and doxorubicin hydrochloride have been selected as model drugs thus hydroxyapatite nanotubes and nanospheres have been chosen as potential drug carriers. In order to incorporate the drug molecules into the mentioned hydroxyapatite nanoforms, the technique of physical adsorption has been used. It was proved by authors that hydroxyapatite nanotubes exhibited higher application potential as drug carriers than hydroxyapatite nanospheres, which probably was caused by their highly porous structure and large specific surface area. Moreover, hydroxyapatite nanotubes, compared to the hydroxyapatite nanospheres, showed better effectiveness in the cell internalization (encapsulation). Another interesting solution concerns the development of hydroxyapatite/alginate gels as carriers of paclitaxel. The spray-drying technique was used by Yoshioka et al. [106] to obtain hydroxyapatite microparticles incorporated with the anticancer drug. Next, such prepared microparticles containing paclitaxel (2.4 and 7.7 wt %) were mixed with sodium alginate. In the next step, the developed reaction mixture was subjected to the gelling process with the use of calcium ions. Finally, the compressive strength of hydroxyapatite/alginate gels and the drug-release profile from such developed materials have been investigated. It was stated that the mechanical properties of these gels depend on both the amount of hydroxyapatite microparticles and the concentration of the alginate. It was observed that the drug release was gradual in the case of the hydroxyapatite/alginate gels. On the other hand, in the case of hydroxyapatite/paclitaxel systems, without the alginate the drug release occurred very rapidly at the beginning of the study. The drug delivery by drug carriers in the form of gels was also reported by Watanabe et al. [107]. They developed the ternary system consisting of hydroxyapatite nanoparticles, the anticancer drug paclitaxel, and the collagen gel. Hydroxyapatite was received by the hydrothermal method and was used as a carrier for water-insoluble paclitaxel. Next, nanoparticles incorporated with the drug have been introduced into a collagen gel. It was reported that the introduction of paclitaxel into the collagen gel may interfere with the drug activity. This is due to the fact that paclitaxel is a strongly hydrophobic drug, and therefore paclitaxel particles may aggregate in collagen gels and finally become inactive. What is important is that the performed investigations indicate that in viewpoint of the effectiveness of the developed ternary system, the stage of the adsorption of paclitaxel to hydroxyapatite was particularly significant.

#### 5.1.3. Conjugates of Collagen/HAp/Vancomycin: Preparation and Application

**Figure 6:**
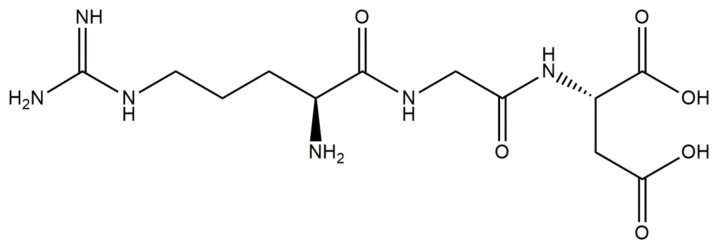
Schematic representation of vancomycin’s chemical structure.

Vancomycin (Figure 6) is a strong glycopeptide antibiotic that acts on Gram-positive bacteria [108]. This drug is indicated for application in patients of all age groups in the treatment of such infections as complicated skin diseases and soft tissue infections [109], community-acquired and hospital-acquired pneumonia [110], bone and joint infections [111], and infectious endocarditis [112]. There are many reports concerning investigations of the use of vancomycin for bone regeneration [113,114,115]. In such a case, an antibiotic is usually combined with hydroxyapatite, which provides an adequate bioactivity [116]. For example, Parent et al. [117] determined the profile of vancomycin release from hydroxyapatite scaffolds that were treated with different concentrations of this antibiotic. In the research, bactericidal and bacteriostatic activity of vancomycin toward *Staphylococcus aureus* and the stability of such systems were also determined. It was reported that the scaffolds with antibiotic could be stored at room temperature for three months and that drug degradation did not occur. Furthermore, all analyzed scaffolds showed good bactericidal properties against *Staphylococcus aureus*, wherein the highest concentration of vancomycin reduced the adhesion of preosteoblast cells. Thapa et al. [118] also carried out studies on vancomycin release. In their research, collagen mimetic peptide tethered to vancomycin and containing liposomes hybridized to hydrogels based on collagen were developed. They determined the in vitro and in vivo antibacterial activity against methicillin-resistant *Staphylococcus aureus* (MRSA). The performed investigations showed the increased antibacterial activity of these materials both in vitro and in vivo. The increased effect was visible even after multiple bacterial inoculations.

The ternary system containing hydroxyapatite, collagen, and vancomycin also seems to be an interesting solution [119,120,121]. For example, the purpose of the research of Egawa et al. [122] was to determine the possibility of local antibiotic administration using the hydroxyapatite/collagen composite as a drug-delivery system. The studies were conducted using eight antibiotics: cefazolin, amikacin, cefotiam, minocycline, daptomycin, teicoplanin, piperacillin, and vancomycin. Among the analyzed drugs, vancomycin showed particularly high adsorption to hydroxyapatite/collagen composites and antibacterial activity up to 14 days after subcutaneous implantation in Wistar rats. In contrast, antibiotics such as cefazolin, piperacillin, and cefotiam exhibited reduced adsorption and lower antibacterial activity. Next, the main research subject of Suchy et al. [123] was to develop the osteoinductive and resorbable layer based on a collagen/hydroxyapatite composite that may be used as a pro-osteointegration interface while simultaneously playing the role of a local drug-delivery system. The kinetics of vancomycin release, the antimicrobial activity, and the cytocompatibility of layers containing the antibiotic have been evaluated. In order to obtain collagen/hydroxyapatite/vancomycin systems, three methodologies have been tested. Two of them consisted of the direct introduction of the antibiotic into the dispersion from which microstructured layers were obtained via lyophilization. Electrospinning was selected as the third method, resulting in the preparation of nanostructured layers. It was proved that the highest concentration of antibiotic released in the longest period of time was provided by materials obtained via the third method; i.e., electrospinning. Importantly, collagen/hydroxyapatite/vancomycin layers obtained by electrospinning did not exhibit cytotoxicity toward osteoblasts.

Coelho et al. [124] have also conducted investigations on the development of the material providing vancomycin release and simultaneously inducing bone regeneration. In their work, they focused on the preparation of porous heparinized nanohydroxyapatite/collagen granules containing vancomycin. Based on the conducted studies, it was stated that the application of heparinized nanohydroxyapatite/collagen granules in the treatment of osteomyelitis returned promising results; i.e., the ability of the release of vancomycin to eliminate bacteria while achieving bone-stimulant properties.

#### 5.1.4. Conjugates of Collagen/HAp/Tetracyclines: Preparation and Application

**Figure 7:**
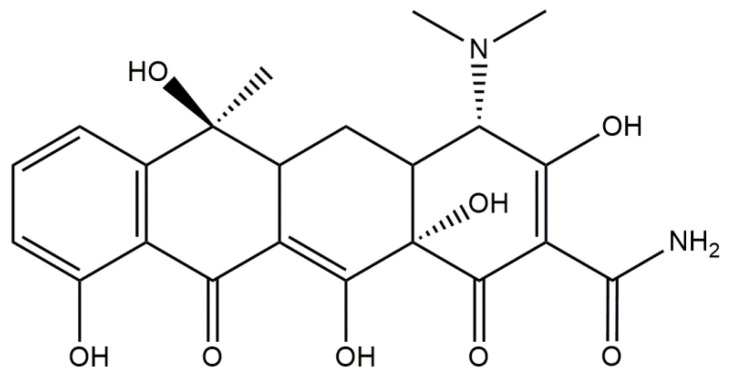
Schematic representation of tetracycline’s chemical structure.

Tetracyclines (Figure 7) are a group of broad-spectrum antibiotics. The mechanism of their action is based on the inhibition of the biosynthesis of proteins by preventing the attachment of aminoacyl-tRNA to the ribosomal acceptor site [125]. These therapeutics exhibit activity against a wide spectrum of Gram-positive and Gram-negative bacteria, as well as against atypical organisms such as mycoplasmas, chlamydiae, rickettsiae, and protozoan parasites [126]. Due to their beneficial antimicrobial properties and the fact that they do not cause any serious side effects, tetracyclines are widely used in the treatments of both human and animal infections [127]. The most frequently mentioned antibiotics belonging to the group of tetracyclines are chlortetracycline [128], tetracycline [129], oxytetracycline [130], doxycycline [131], and minocycline [132]. Drugs from this group are used for treatment of acne and rosacea, as well as for urinary tract infections, Lyme disease, and nonspecific respiratory-tract infections [133].

The development of the field of bone-tissue engineering indicates numerous studies on the combination of tetracycline antibiotics with hydroxyapatite [134,135]. Ternary systems consisting of such an antibiotic, hydroxyapatite, and collagen also have been investigated. For example, Rusu et al. [136] synthesized composite materials based on collagen/carboxymethylcellulose (CMC)/hydroxyapatite incorporated with tetracycline. The synthesis of such composites was divided into two steps: the first involved the mineralization of collagen/carboxymethylcellulose gel, while the second one consisted of incorporation of tetracycline into such formed gel. In the first step, collagen gel and carboxymethylcellulose were homogenized in a collagen:CMC = 2:1 mass ratio. Next, the mineralization process was conducted using Ca(OH)_2_ and NaH_2_PO_4_ ⋅H_2_O as hydroxyapatite precursors. In the next step, the previously formed system was cross-linked with 1% glutaraldehyde solution. Incorporation of the composite with tetracycline was performed by absorption of the adequate amount of tetracycline hydrochloride (0.5 g tetracycline/10 g composite) as an aqueous solution. The resulting composite was characterized using such techniques as FT-IR spectroscopy, UV-VIS spectrophotometry, and SEM. Based on the conducted investigations, it was stated that the developed system provides long-term tetracycline release, ensuring at least 6 days of antibacterial properties.

The next compound belonging to the group of tetracycline antibiotics that also has an application in the preparation of HAp/collagen materials and is more commonly applied than tetracycline is doxycycline [137]. A combination of hydroxyapatite, collagen, and doxycycline has found broader application than a combination of HAp/collagen with tetracycline [137]. For example, the main objective of Semyrari et al. [138] was to develop composite scaffolds with both antibacterial and osteoinductive properties for the repair of bone defects. Hydroxyapatite nanoparticles containing doxycycline (10 wt %) were obtained by the wet precipitation method and subsequently introduced into the collagen solution. Scaffolds were prepared by the freeze-casting method. Next, numerous studies were aimed at comparing the activity of scaffolds with and without the addition of the antibiotic. Based on the in vivo tests in Wistar rats, the beneficial impact of the scaffold containing doxycycline on the bone-healing process was reported. What is important is that histopathological evaluation exhibited that free spaces in tested collagen/hydroxyapatite/doxycycline scaffolds were fully replaced by the newly formed bone tissue. Mederle et al. [139] also performed investigations on the preparation of innovative materials based on collagen and hydroxyapatite containing doxycycline. These biomaterials were obtained in spongious forms as a result of the lyophilization process. Subsequently, their water sorption ability, morphology, and drug-release profile were examined. It was stated that the higher amount of hydroxyapatite resulted in the more compact structure of obtained composites, their lower sorption properties, and a more effective drug release.

The combinations of hydroxyapatite, collagen, and minocycline are also the subject of many studies. For example, Martin et al. [140] developed scaffolds based on poly(lactic acid) (PLA) with multicomponent coatings on their surfaces. The main task of such a coating is to reduce bacterial biofilm formation while promoting the activity of human mesenchymal stem cells (hMSC) derived from bone marrow. For this purpose, PLA scaffolds were prepared using 3D printing and subsequently subjected to the multifunctionalization process. Collagen (coll), minocycline (MC), and citrate-hydroxyapatite nanoparticles (cHA) were used. It was stated that PLA-coll-MC-cHA scaffolds provided structural similarity to natural bone and ensured good compressive strength. Furthermore, based on the drug-release profile, it was proved that the release of minocycline was adequate toward the therapeutic application; i.e., it was intense enough to show antibacterial activity against the formation of *S. aureus* biofilm, and at the same time did not exhibit a cytotoxic effect in relation to hMSC. Additionally, it was stated that the presence of cHA on the surface of such a scaffold led to the more intense proliferation and the osteogenic differentiation of cells.

#### 5.1.5. Conjugates of Collagen/HAp/Gentamicin: Preparation and Application

**Figure 8:**
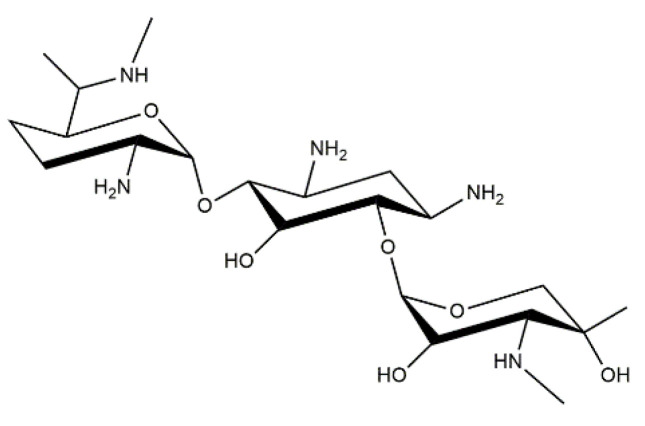
Schematic representation of gentamycin’s chemical structure.

Gentamycin (Figure 8) is an aminoglycoside antibiotic with bactericidal activity against Gram-negative bacteria [141]. Although there are many literature reports concerning the resistant strains of Gram-negative bacteria, the majority of these microorganisms that show facultative or aerobic metabolism are susceptible to gentamycin. Therefore, this drug is considered as a good solution in the treatment of many common infections [142]. The U.S. Food and Drug Administration (FDA) approved its application in the treatment of infections caused by such bacteria as *Klebsiella pneumoniae*, *Serratia marcescens*, *Escherichia coli*, *Citrobacter* spp., and many others [143]. Due to its wide bactericidal activity, gentamycin is applied in the treatment of the bacterial sepsis in newborns [144] and also in the case of ventilator-associated pneumonia [145]. The described drug is usually administered parenterally due to its minimal absorption from the gastrointestinal tract. Its use is unfortunately limited due to potential serious side effects, most often ototoxicity and nephrotoxicity. Gentamycin is also used as an additive to such medical materials for prosthetic-joint-associated infections or cement spacers [146]. In recent years, a great interest in the so-called drug-delivery systems has been observed. Many investigations are performed to develop an effective delivery system for a wide area of active substances such as gentamycin [147].

It is worth noticing that a growing interest in multicomponent systems containing gentamycin and other antibiotics, hydroxyapatite, and collagen is currently observed. Such systems aim at the formation of the material providing the stable release of antibiotics while supporting bone-tissue growth [148,149]. For example, Ionescu et al. [150] performed the synthesis of biocomposite materials consisting of hydroxyapatite, gentamycin, and collagen. In the first step, a polymer matrix with hydroxyapatite was obtained. Subsequently, collagen/hydroxyapatite composite was immersed in the gentamycin solution for 48 h to attach the drug via chemisorption. During the analysis of the composites received, the main focus was on determining the antibiotic release profile. It was reported that the introduction of gentamycin in the collagen/hydroxyapatite composite resulted in an increase in the therapeutic effect of the drug due to the possibility of its slow release over a long period of time. Next, the main objective of the research of Suchy et al. [151] was to develop biodegradable coatings based on collagen, hydroxyapatite nanoparticles, vancomycin hydrochloride, gentamycin sulphate, and their combination. The developed material could potentially be used for the treatment of prosthetic joint infections, as well as for the prevention of infections that might occur in the case of joint-replacement procedures. Collagen/hydroxyapatite coatings containing various concentrations of hydroxyapatite were subjected to analyses aimed at the determining the kinetics of the in vitro antibiotic release, antimicrobial activity, and cytotoxicity toward SAOS-2 bonelike cells. It was reported that collagen/hydroxyapatite coatings released high concentrations of gentamycin and vancomycin for 21 days. Additionally, the effective activity of such materials against bacterial isolates was also confirmed. Importantly, the results obtained showed that the presence of hydroxyapatite in tested collagen coatings did not affect the kinetics of the vancomycin release, but on the other hand, simultaneously lower gentamycin release from such coatings was observed. It was also reported that the combination of vancomycin and gentamycin in collagen/hydroxyapatite coatings showed adequate cytocompatibility toward SAOS-2.

#### 5.1.6. Conjugates of Collagen/HAp/Alendronate: Preparation and Application

Repair of large-size bone defects remains challenging, as it is critical to develop scaffolds that both chemically and structurally mimic the native extracellular matrix (ECM) of bone. Bone is an unique triphasic tissue composed of cells (hydrated extracellular organic matrix—mainly type I collagen (Col); and an extracellular mineral phase—mainly hydroxyapatite (HAp)).

**Figure 9:**
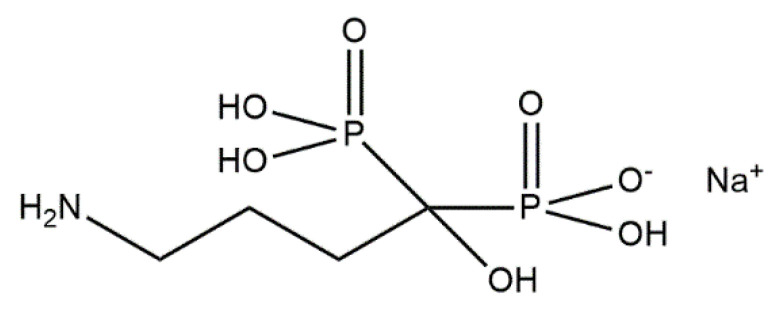
Schematic representation of alendronate sodium’s chemical structure.

Alendronate (AL) (Figure 9) is a bisphosphonate (BP) drug used to prevent the loss of bone density (e.g., to treat osteoporosis). The two phosphonate groups present in the structure of this compound are essential both for binding to hydroxyapatite (HAp) and for the biochemical mechanism of action. Experts caution that the benefits of prolonged use of BPs must be carefully weighed against the potential negative effects of oversuppression of bone metabolism, which is why the development of strategies for local administration of BPs have become interesting. One attempt is the direct synthesis of HAp crystals modified with BPs, because BPs strongly inhibit mineralization and have been used as anticalcification agents. Bigi and Boanini have carried out a classical method of synthesis of HAp in an aqueous medium, wherein alendronate was added to the phosphate solution before dropping it into the calcium solution. Following this synthesis, the precipitated hydroxyapatite with unique crystalline phase contained incorporated AL in a content up to about 7 wt%. The authors proved the precipitation of amorphous calcium alendronate [152]. Their further in vitro study demonstrated that alendronate was able to promote osteoblast activation and extracellular matrix mineralization without collagen degradation, and to inhibit osteoclast proliferation [153]. Moreover, alendronate–hydroxyapatite thin films deposited by matrix-assisted pulsed laser evaporation (MAPLE) displayed the downregulatory role of alendronate on the inflammatory reaction [154].

For guided bone regeneration, numerous scaffolds have been developed based on natural or synthetic polymers. Among them, hydrogels improved by physical, enzymatic, or chemical cross-linking have been proposed. Ma et al. [155] developed a biomimetic hybrid hydrogel composed of hydroxyapatite (HAp), collagen, and alendronate (AL) reinforced by cross-linking via genipin (GNP) for the application as bone-tissue-engineering scaffolds. Synthesis first included preparation of a HAp-AL system by separately dissolving them in NaOH solution, then mixing and stirring, followed by their isolation by extensive dialysis and freeze-drying. The synthesis route of the hydrogels included separately dissolving collagen and GNP in PBS and dispersing HAp-AL in the collagen/PBS solution, and then such obtained mixtures were crosslinked by GNP. The obtained collagen/HAp-AL hydrogels indicated decent biocompatibility and promoted the adhesion and proliferation of MC3T3-E1 cells, proving the biomimetic nature of these hybrids, which include major components of native bone tissues.

Sugata et al. [156] evaluated the effect of AL on bone formation in the presence of a porous HAp/collagen implant using a rabbit model. HAp/Col nanocomposite fibers were synthesized from atelocollagen derived from porcine skin, Ca(OH)_2_, and H_3_PO_4_ using a coprecipitation method described previously. The HAp/Col fibers were lyophilized to prepare porous HAp/Col implants, cross-linked by thermal dehydration at 140 °C under vacuum, and sterilized by irradiation. The HAp/Col implants were inserted into a defect produced in the condyles of 72 rabbits, while AL was injected once a week. AL administration suppressed the resorption of implants, and lowered the mineral density of newly formed bones. Their study suggest to suspend the administration of BPs after the implantation of HAp/Col (or other bioabsorbable bone substitutes) until the implants are resorbed.

### 5.2. Conjugates of Collagen/HAp/Metals and Nanoparticles

Metallic materials are characterized by very good mechanical parameters, which are better than ceramics or polymers, and therefore they are used in tissue engineering. However, most metallic materials exhibit lower biocompatibility than, for example, ceramics, which may cause blood clots and allergic reactions. For this reason, metals are often used in tissue engineering as alloys or metal–ceramic or metal–polymer compositions [157]. In addition, nanomaterials are very popular in biomedical applications. The unique properties of nanoparticles, including a high ratio of surface area to volume; antimicrobial activity; physical, mechanical and biological properties; and their unique size, have made them widely used in medicine and pharmacy. When selecting nanoparticles for tissue-engineering applications, the chemical, physical and biological aspects of nanostructures have been taken into account [158,159,160].

#### 5.2.1. Conjugates of Collagen/HAp/Cisplatin and Platinum Nanoparticles: Preparation and Application

**Figure 10:**
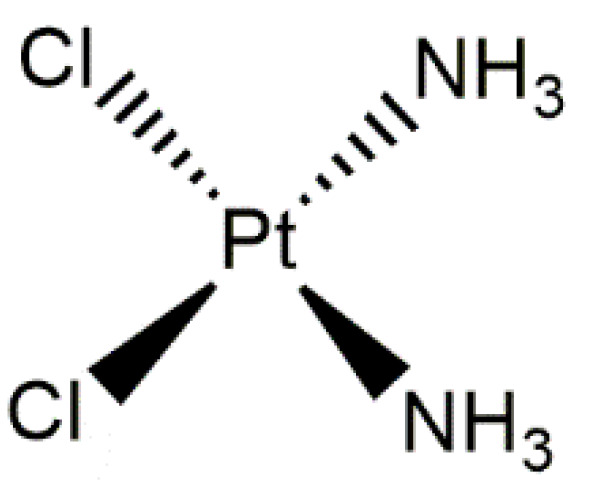
Schematic representation of cisplatin’s chemical structure.

Cisplatin (also known as cisplatinum or cis-diamminedichloroplatinum (II)) (Figure 10) is widely used as an anticancer drug. It finds application in many cases of malignant tumors such as cancer of the testicles, ovaries, neck, large intestine, lungs, or bladder [161]. The effect of cisplatin mainly involves the damage of the DNA of cancer cells. Furthermore, this anticancer drug also results in the cytoplasmic dysfunction of organs, particularly in the case of the endoplasmic reticulum and mitochondria. It also activates the apoptotic pathways and causes cell damage via oxidative stress and inflammation [162]. One of the factors that limits the use of cisplatin is its toxicity to the kidney. A common side effect that occurs during the treatment with this cytostatic is acute kidney damage [163]. Furthermore, other side effects include allergic reactions, reduced resistance to infections, hearing loss, or gastrointestinal disorders [164]. Additionally, a certain limitation is also the fact that cisplatin treatment often leads to the appearance of so-called chemoresistance, which in turn contributes to the failure of the treatment [165].

Due to the numerous limitations in the application of cisplatin, its new combinations, as well as new methods of its delivery to cancer cells, are currently the research subject of many investigations [161,166]. Additionally, as was previously mentioned in the subsection concerning paclitaxel, the simultaneous application of an anticancer drug (here, cisplatin) and hydroxyapatite positively affects both the cancer treatment and bone regeneration. For example, studies on the release of cisplatin from the developed carrier have been performed by Barroung et al. [167]. In their work, the characteristics of cisplatin binding by the suspensions of synthetic hydroxyapatite crystals in the aqueous environments was described. It was stated that the cisplatin adsorption via hydroxyapatite suspension significantly depended on the ionic composition of the solution in which this process took place. It was also observed that at a constant pH of 7.4, much more cisplatin was adsorbed by the hydroxyapatite crystals in the chlorine-free phosphate solution or in Tris buffer than in phosphate-buffered solution containing chloride ions. What is important is that the amount of cisplatin bound with hydroxyapatite and desorbed to the tested solution was also gradually increased depending on the increasing concentration of chloride in the equilibration solution. Palazzo et al. [168] have also conducted studies on the adsorption and desorption of cisplatin from hydroxyapatite. In their investigations, nanocrystalline hydroxyapatite with platelet or needle morphologies were analyzed. The following drugs were tested: cisplatin and di(ethylenediamineplatinum)medronate (new platinum (II) complex). It was reported that the adsorption and desorption kinetics depended on the specific properties of the particular drugs and the morphology of the hydroxyapatite nanoparticles. Moreover, it was proved that the adsorption of both analyzed platinum complexes was driven by the electrostatic forces. What is important is that adsorption of the positively charged cisplatin species was more favored on the phosphate-rich surface of hydroxyapatite needles. On the other hand, adsorption of negatively charged alendronate was more promoted on the surface of the calcium-rich hydroxyapatite platelets.

Andronescu et al. [169] have developed material for bone regeneration that aimed at fulfilling two important functions (1) the replenishment of the missing bone that is a result of surgery to remove bone cancer, and (2) the local administration of cisplatin to the other cancer cells. For this purpose, a novel cisplatin-loaded collagen/hydroxyapatite composite was proposed. Such a material was designed so that the collagen-to-hydroxyapatite weight ratio was similar to the one occurring in the bone composition. These composite materials were prepared using collagen gel (collagen type I) and Ca(OH)_2_ and NaH_2_PO_4_ as hydroxyapatite precursors. Biological investigations performed to evaluate the anticancer activity of cisplatin in such formed collagen/hydroxyapatite composite materials toward osteosarcoma G292 cell lines showed cytotoxic and antiproliferative effects of the tested composites, depending on the concentration of cisplatin released to the injury site. In turn, Ficai et al. [170] constructed collagen/hydroxyapatite–magnetite–cisplatin composites via a layer-by-layer technique. The composites obtained differed in the hydroxyapatite content. The anticancer effect of these composites toward the cell line derived from cervical cancer was determined. It was reported that the materials with higher hydroxyapatite content showed greater anticancer activity due to the better cisplatin adsorption that led to the higher amount of the active substance in the matrices. Furthermore, the cytotoxicity of the tested composites depended on the cisplatin content and the number of layers of composite material.

#### 5.2.2. Conjugates of Collagen/HAp/Magnetite Particles (Iron Oxide Nanoparticles): Preparation and Application

Along with the currently observed development of nanotechnology, the appearance of various magnetic colloidal particles with a nanometric size has been denoted [171]. A great interest in this research topic directly translates into the wide application of such particles in biomedicine, e.g., in cancer treatment, drug delivery, or diagnostics [172,173]. Particular attention is directed toward the iron oxide that is widely applied in systems of controlled drug release [174]. Due to its superparamagnetic properties and large specific surface area, this compound exhibits relatively easy binding to natural biomolecules or drugs [175]. The combinations of hydroxyapatite with these nanoparticles in two-component systems [176,177] or more popular three-component systems containing also an adequate biopolymer have been widely examined [178]. For example, Heidari et al. [179] developed the method of preparation of iron oxide nanoparticles inside chitosan/hydroxyapatite scaffolds. Hydroxyapatite and chitosan were prepared by extraction from bovine bone and the exoskeletons of shrimps, respectively. In order to obtain the composites containing magnetic nanoparticles, FeCl_2_ × 4H_2_O and FeCl_3_ × 6H_2_O were added to 2% acetic acid solution, and stirred intensely for 30 min. Next, hydroxyapatite powder and chitosan were introduced into such formed solution. After obtaining the homogeneous mixture, it remained for 12 h and was placed in the appropriate form. After drying, the scaffolds received were subjected to such investigations as FT-IR spectroscopy and scanning electron microscopy (SEM). Additionally, the use of a particle-size analyzer and transmission electron microscopy (TEM) allowed the authors to determine the average size of the iron oxide particles present inside the scaffold. The size of particles was 10–40 nm. Magnetic measurements showed that the saturated magnetic intensity (Ms) was approx. 3.04 emu/g.

Interesting investigations have been also carried out by Zaborowska et al. [180]. They synthesized nanocomposite scaffolds based on bacterial cellulose (BC), magnetic nanoparticles, and hydroxyapatite nanoparticles. Physicochemical studies on such systems showed homogeneous distribution of both hydroxyapatite and iron oxide nanoparticles in the BC matrix. The presence of HAp and iron oxide nanoparticles affected the increase in the porosity degree, with a simultaneous decrease in the crystallinity degree of such composites compared to the matrix without nanoparticles. Such an impact of introduced nanoparticles may provide an adequate biodegradability of such modified scaffold when applied in bone regeneration. A cytotoxicity assay indicated that the scaffold did not exhibit toxicity toward L929 murine fibroblasts. Furthermore, an in vitro biocompatibility examination exhibited good adhesion and the differentiation of bone cells on the surface of the tested composites.

Next, Andronescu et al. [181] developed a composite material based on collagen and hydroxyapatite containing various additional concentrations of magnetic nanoparticles. The main objective of the application of the mentioned nanomaterials was the possibility of the use of such formed systems in controlled hyperthermia activated by an electromagnetic field. First, the mineralization of collagen was performed. For this purpose, calcium hydroxide suspension and sodium dihydrogen phosphate solution were added to the collagen gel for precipitation of hydroxyapatite. Next, an adequate amount of magnetic nanoparticles (obtained previously by the coprecipitation method using FeCl_3_ and FeSO_4_ as precursors of iron ions) was introduced into the previously formed suspension. The obtained composites were freeze-dried and subsequently subjected to numerous investigations. It was stated that the coll/HAp/magnetic nanoparticles composites containing 1 and 2 wt % iron oxide nanoparticles were not adequate for application in hyperthermia, in contrast to composites with the content of these nanomaterials at 5 wt % (activation time—20–30 min at 150 Hz).

#### 5.2.3. Conjugates of Collagen/HAp/Carbon Nanotubes: Preparation and Application

Carbon nanotubes (CNTs), with their high aspect ratio (length to diameter), high strength, and stiffness, have potential to enhance mechanical properties of biocomposites. Recent studies suggest that they also show bioactive properties that stimulate bone regeneration. A composite material composed of HAp/collagen/CNTs combines properties of each ingredient; i.e., HAp provides osteoconductivity and biocompatibility, collagen provides plasticity and increases biocompatibility, and CNTs enhance mechanical properties and affect the dynamics of drug release.

CNTs are rolled graphene sheets with hemispherical endcaps. Single-walled (SW) NTs contain just one graphene sheet, while multiwalled (MW) NTs are composed of many sheets with an interlayer distance of ~0.34 nm. SWNTs have a size up to 2 nm in diameter and several micrometers in length, while MWCNTs range from 2 to 100 nm in diameter and up to few millimeters in length. CNTs have excellent mechanical properties due to the strength of their carbon sp2 bonds. The tensile strength of MWCNTs measured experimentally range from 11 to 63 GPa, and the Young’s modulus was estimated at 270 to 950 GPa [182]. The mechanical behavior of CNTs in the composites may differ adequately with the interfacial bonding between the phases. Literature reports suggest that SWNTs have a higher aspect ratio and greater surface than MWCNTs, allowing for more interfacial bonding and serving as better fillers [183], but on the other hand, it is difficult to synthesize them in larger quantities. Thostenson et al. [184] reviewed CNTs composites and found that the mechanical properties of the composite material can be still enhanced regardless the type of CNTs or the synthesis method.

A serious concern for in vivo applications is the toxicity of CNTs, while studies reporting on biological and toxicological properties are contradictory. However, the toxicity of CNTs has been shown to be reduced through chemical functionalization or coating with substances like polymers, hydroxyapatite, or collagen [185]. Zanello et al. [186] found that rat osteosarcoma cells grew best on the surface of as-prepared SWCNTs, while the lowest growth was found on the MWCNTs, suggesting that the cell shape and cell differentiation can be controlled by the use of SWCNTs or MWCNTs. Their findings also indicated that the CNTs could induce the formation of type I collagen by fibroblasts and osteoblasts. Collagen–CNT composite materials sustained high smooth muscle cell viability, and CNTs in suspension in the culture medium were incorporated into the cell cytoplasm by macrophages and leukemia cells without affecting the cell population growth [187].

Preparation of the HAp/collagen and CNT composite needs to be performed so as to ensure homogenous dispersion of CNTs in the solvent and encourage the interaction between them and the HAp/collagen matrix. This is practically impossible without chemical functionalization of the CNTs, which tend to agglomerate and are insoluble in water or any organic solvents. Several reviews have covered this problem [188,189], and summarized that chemical oxidation was by far the most common method. Typical oxidizers include HNO_3_, H_2_SO_4_, KMnO_4_, OsO_4_, and RuO_4_. This method covalently attaches a wide range of functional groups to the CNTs’ ends and existing wall defects. The more defects (which also can be induced by sonication with acid) are present in the structure of CNTs, the more sites for functional group attachments, which on the other hand cause their cleaving and cutting into shorter segments, and as a result weaken them mechanically [190]. An alternative is noncovalent functionalization (e.g., with sodium dodecylsulfate—SDS) or coating the CNTs with a polymer (e.g., phenol resin), which are less damaging and very effective in increasing the solubility and enabling CNTs to interact with the matrix material. This method weakens the hydrogen bonding, π–π stacking, electrostatic forces, and van der Waals forces, as well as hydrophobic and hydrophilic interactions. This type of functionalization can attach collagen to sidewalls or wrap HAp chains around the CNTs.

Several methods can be used to disperse functionalized CNTs in a matrix, e.g., physical blending, shear mixing, ball milling, and dry mixing of HAp powders and MWCNTs, which is commonly used with polymer–CNTs composites. In situ formation involves synthesizing the matrix material around the CNTs. In several papers, synthesis of HAp via a precipitation reaction in the presence of MWCNTs has been reported. This in situ method gives the CNTs a better opportunity to interact with CA^2+^ and PO_4_^3−^ ions as the HAp is nucleating, resulting in more homogeneous composites, and is a simpler procedure compared with mixing [191]. The biomimetic mineralization method is used to synthesize bone-like apatite under ambient conditions in aqueous environments. It is possible to induce the growth of HAp crystals on graphene immersed in an unstable solution with phosphate ions and calcium ions concentrations similar to simulated physiological conditions (simulated body fluid (SBF) solution). This mineralization process strongly improves crystallization of HAp [192].

Jing et al. [193] made use of collagen and hydroxyapatite as substrates in order to mimic the extracellular matrix of bone, and took advantage of functional MWCNTs, aiming at designing a novel three-dimensional porous biocomposite. The preparation included acid treatment of CNTs (functionalization), their dispersion in deionized water, and embedding in collagen slurry. After 1 h of blending of such a slurry at 4 °C, NaH_2_PO_4_ and CaCl_2_ were added, and the whole mixture was stirred for 2 h. To initiate gelation, the mold with mixture was incubated (37 °C, 24 h), and after the incubation the gel obtained was freeze-dried at −80 °C. Lyophilized scaffolds were three-dimensional, porous, and foamy in structure, and showed high stiffness—approximately 10-fold stiffer than Col/HAp. CNTs/Col/HAp scaffolds were superior in promoting bone marrow mesenchymal stem proliferation. Moreover, in vivo repair of the rat calvarial defects using this composite showed new bone formation.

Türk et al. [194] developed and characterized highly porous collagen/functionalized multiwalled carbon nanotube/chitosan/hydroxyapatite (Col/MWCNTs/CS/HAp) bone scaffolds. The Col/MWCNTs/CS composites were fabricated first by freezing (−40 °C at 0.9 °C/min) and lyophilization (48 h, 0 °C, and 200 mTorr) and subsequent biomineralization of HAp on the fabricated scaffolds in concentrated SBF solution by an in vitro biomimetic method. The produced porous bonelike scaffolds exhibited increased strength (from 524 to 1112 kPa), hydrophilicity (from 87.8° to 76.7°), and good physiological solution (PBS) absorption.

E. da Silva et al. [195] described a collagen/carbon nanotube composite treated with mineralized hydroxyapatite crystals (mineralization induced in vitro and proceeded under physiological conditions). SWCNTs were prepared by the arc-discharging method, and collagen in turn was extracted from rat tails. Their results indicated that the nanostructure surface enabled the formation and increased induction of HAp crystals. Moreover, the increased mechanical rigidity, combined with the enhanced nanostructured character of the composite, improves its performance.

#### 5.2.4. Conjugates of Collagen/HAp/Graphene: Preparation and Application

Graphene and its derivatives, such as graphene oxide (GO) and reduced graphene oxide (rGO), feature two-dimensional nanosheets of hexagonally bonded carbon atoms, with a large surface area, high conductivity, and strong mechanical properties with high elasticity, flexibility and biocompatibility [196]. These advantages promote using graphene-based composites in bone repair or regeneration, as they additionally induce osteogenesis and chondrogenesis [197].

In order to prepare graphene/HAp bulk composites or coatings with significantly improved mechanical properties, various preparation techniques using special equipment have been exploited, such as thermal spraying techniques, hydrothermal synthesis, spark plasma sintering, or hot isostatic sintering [198,199].

HAp can be also synthesized onto graphene and its derivatives using in situ synthesis methods. Usually, graphene-based powders are first dissolved and exfoliated in deionized (DI) water by ultrasonic dispersion to obtain an uniform solution; then Ca(NO_3_)_2_ is added into the graphene-based solutions by stirring for a desired time; afterward, the pH of the suspension is adjusted to 10 using ammonia water, and (NH_4_)_2_HPO_4_ is added dropwise into the mixture. The resulting composite solution is aged for days to ensure the full transformation of apatite into hydroxyapatite and good crystallinity [200]. This method increases the interfacial bonding strength between graphene and HAp, facilitating the stress transfer from the matrix to the graphene-based nanofillers.

GO can be also biofunctionalized by peptide, chitosan, or collagen to improve the mineralization process. In this case, the in situ method can be also used for the synthesis of hydroxyapatite on colloidal collagen-graphene composites [201,202]. Yadav et al. [201] revealed the coexistence of both graphene (G) and HAp in the lattice and, moreover, demonstrated an interaction between exfoliated graphene, collagen, and hydroxyapatite through pyridinic N-oxide moiety. All other characterizations lead to the conclusion that the more the graphene content (1–3 layers), the better the composites’ properties in the view of in vitro antibacterial tests, the sensitivities of the microorganism species, and the stability studies in SBF. Results clearly indicated that these composites could find applications in the biomedical industry particularly as air filters. Zakharov et al. [202] used an in situ method to synthetize hydroxyapatite/graphene oxide/collagen nanohybrid composites with tailored nanocrystalline hydroxyapatite (NCHAp), with sizes and morphology for bone implants.

GO and collagen are promising materials for tissue regeneration, and are particularly interesting for a novel biomimetic mineralization route employing a graphene oxide (GO)–collagen (COL) conjugate as a template material for the biomineralization of hydroxyapatite (HAp) [192]. For example, Wang and coworkers reported the synthesis of GO-incorporated collagen/HAp composites for bone-repair applications using biomimetic mineralization in order to better mimic natural bone [203]. Particularly, preparation of a COL/HAp/GO composite by biomimetic mineralization first requires the obtaining of a COL/GO solution, then a mass fraction of COL/GO scaffolds, then freeze-drying and immersion in cross-linking solution (ribose, acetone, ammonia). The COL/GO scaffolds after immersion were freeze-dried again. The prepared cross-linked COL/GO composites were irradiated with gamma rays and immersed in SBF solution for 3 days at 37 °C. After mineralization, the samples were taken out, washed with distilled water, and freeze-dried to remove moisture [204]. The results showed that the GO and nHAp were dispersed homogeneously in the composite. Additionally, COL/GO/nHAp composites had a high porosity. By increasing the GO content from 0 to 4 wt %, the COL/GO/nHAp composites exhibited improved hydrophilic and mechanical properties. The cell viability and proliferation results showed that osteoblastic cells could adhere and develop on COL/GO/nHAp composites. An outstanding proliferation potential was displayed by the cells in contact with COL/GO composites containing 4 wt % nHAp. The GO-reinforced COL/nHAp composites with high mechanical and bioactive properties are potential candidates for bone-tissue engineering.

Graphene/HAp mixed with polymers are often prepared by lyophilization; e.g., Liang et al. [205] reported on hydroxyapatite/collagen/poly(lactic-co-glycolic acid)/graphene oxide (nHAC/PLGA/GO) composite scaffolds containing different amounts of GO that were fabricated by the freeze-drying method. Their results showed that hydrophilicity reinforced their mechanical strength. The Young’s modulus of the 1.5 wt % GO-incorporated scaffolds was greatly increased, while the in vitro experiments proved their excellent cytocompatibility and bone-regeneration ability.

A novel implant coating material containing graphene oxide (GO), collagen (COL), and hydroxyapatite (HAp) was fabricated by electrodeposition by Yılmaz et al. [206]. HAp, HAp/GO, and HAp/GO/COL coatings on the surface of the Ti16Nb alloy formed a corrosion barrier layer. For the HAp/GO/COL coating, the highest corrosion resistance, hardness, and elastic modulus (compatible with cortical bone) were obtained due to the compactness and homogeneity of the coating structure. This can also increase wear resistance for load-bearing implants. The contact angles of the coated samples were close to 0°, wherein the increased surface wettability is important for cell adhesion. The addition of collagen to the HAp/GO coating increased the cell adhesion and viability of 3T3 fibroblast cells.

For in vivo applications, careful consideration of the biocompatibility and toxicity of CNTs and graphene nanosheets is important. To date, few studies have been done on biocompatibility of graphene [196] and CNTs [207]. Graphene in the form of nanowalls effectively damages the cell membrane of the bacteria due to the very sharp edges of nanowalls, resulting in their inactivation [208]. Graphene oxide generally is more biocompatible, especially in the forms of nanosheets and nanoflakes [207,209,210]. Graphene and its derivatives incorporated with metals, polymers, and minerals showed promoted mechanical properties and bioactivity in most cases [211]. Bone cell proliferation also has been reported for several carbon nanomaterial composites, including those prepared by coating substrates such as collagen, hydroxyapatite, or polymers [212].

#### 5.2.5. Conjugates of Collagen/HAp/Silver Nanoparticles: Preparation and Application

Silver nanoparticles belong to the group of particles with a size below 100 nm, and are formed mainly by physical or chemical methods. In recent years, particular attention has been directed toward the biological methods of their preparation that are considered as ecofriendly and less toxic compared to other applied methods [213]. The size and antibacterial properties of silver nanoparticles contribute to the wide area of possible applications of these nanomaterials [214]. The areas in which the above-described particles are considered as useful are catalysis, electronics, optics, environmental protection, and medicine [215]. Great application potential of nanosilver is noticed particularly in medicine and the relative areas in which these are applied in diagnostics, as components of coatings of medical devices or as drug carriers [216,217].

Ternary systems based on collagen, hydroxyapatite, and silver have been proposed by Predoi et al. [218] Silver-doped hydroxyapatite has been prepared via a coprecipitation at room temperature. Calcium nitrate and ammonium hydrogen phosphate have been used as HAp precursors, and silver nitrate has been used as a source of silver. Ag-doped HAp has been combined with various amounts of collagen gel (type I fibrillar collagen in the form of gel extracted from calf skin was used). The final materials were obtained via cross-linking performed using 0.5% glutaraldehyde and lyophilization. The purity, crystallinity, and phase composition were determined by the X-ray diffraction (XRD) technique. The presence of functional groups characteristic for HAp was verified using FT-IR spectroscopy. The size of HAp particles and their distribution in the collagen were determined by scanning electron microscopy (SEM). Importantly, the antibacterial activities of the obtained materials against *Escherichia coli* (Gram-negative bacteria) and *Staphylococcus aureus* (Gram-positive bacteria) also were evaluated. In both cases, an increase in the inhibition zone with an increasing concentration of the silver in the tested material was demonstrated. Due to the simplicity of the synthesis methodology applied, the preparation of a homogeneous distribution of Ag-HAp in the collagen, as well as the antibacterial activity of obtained materials of such conjugates, have been considered as interesting and promising for application in the regeneration of bone tissue [218].

A similar combination has been also presented by Ciobanu et al. [219]. The Ag-doped Hap also was prepared by the coprecipitation method and added to the collagen gel. Such mixture was mixed for 24 h and dried at room temperature. Next, XRD and FT-IR techniques were used to characterize the chemical structure and the crystallinity of the final materials. Next, the morphology of the materials and their elemental composition were determined via scanning electron microscopy. The results of the XRD and FT-IR analyses showed the typical structure of hydroxyapatite. It was also proved via the SEM technique that the collagen affected the Ag-doped HAp morphology by increasing the porosity of the final compositions.

#### 5.2.6. Conjugates of Collagen/HAp/Gold Nanoparticles: Preparation and Application

Gold nanoparticles, due to their unique photophysical properties, play an essential role in the development of nanomedicines [220]. Particular attention is paid to the application of these nanomaterials in diagnostics [221]. The application of the described nanoparticles as a tool for the diagnostics of various types of cancer or as systems for the delivery of cytostatics should be also mentioned [222,223]. Furthermore, gold nanoparticles applied in combination with antibiotics cause an increased antimicrobial activity while reducing the need for the high doses of the drugs. Therefore, it is considered that gold nanoparticles playing a role of carriers of antibiotics constitute a promising strategy in antibiotic-based treatment [224,225]. Moreover, the combination of nanogold with hydroxyapatite that forms systems aimed at the improvement of bone-tissue regeneration or at the delivery of drugs (mainly antibiotics) is also known [226,227,228].

For example, Liang et al. [229]. focused in their work on the development of the combination of gold nanoparticles with hydroxyapatite nanoparticles to design new materials affecting the differentiation of human mesenchymal stem cells (hMSCs) into osteoblasts. Such a process may provide rapid bone regeneration and reconstruction. In the mentioned investigations, the biocompatibility of gold nanoparticles/hydroxyapatite nanoparticles systems and the osteogenic induction effect in human mesenchymal stem cells were evaluated. The results of the research confirmed the successful synthesis of hydroxyapatite/nanogold nanocomposites, which exhibited cytocompatibility and caused a synergistic effect on the acceleration of the osteogenic differentiation of human mesenchymal stem cells. Another interesting solution involves the combination of hydroxyapatite and gold nanoparticles with antimicrobial peptides. Such a designed hydroxyapatite/nanogold/arginine nanocomposite consists of (1) hydrophobic gold nanoparticles; (2) positively charged hydrophilic molecules of arginine, which functionalize the surface of gold nanoparticles; and (3) hydroxyapatite, which plays the role of a bioactive carrier of gold nanoparticles with functionalized surfaces. Such a developed nanocomposite provides all favorable properties of antimicrobial peptide with an additional increase in the stability of such formed system due to the presence of gold nanoparticles in combination with hydroxyapatite [230].

Next, Mondal et al. [231] constructed a gold nanoparticle/hydroxyapatite composite using microwave-assisted facile rapid synthesis technology. Such a procedure allows the incorporation of gold nanoparticles onto the surface of hydroxyapatite. Subsequently, the obtained nanocomposites were coated with the collagen biomaterials and analyzed in viewpoint of the drug-loading efficiency, wherein doxorubicin was used as a model drug. It was reported that the maximum drug-loading and drug-release efficiency occurred for a gold/hydroxyapatite/collagen nanocomposite with a concentration of 0.1 wt %. Next, Aryal et al. [232] performed investigations on the development of the material with an application potential in bone-tissue regeneration. In the first step of their research, the collagen was immobilized on gold nanoparticles via an in situ chemical reduction. Then, hydroxyapatite was synthesized with adequate precursors and using a collagen/gold nanocomposite matrix. After the microscopic and crystallographic analyses, it was reported that gold nanoparticles formed a matrix with collagen, which positively affected the hydroxyapatite growth. Therefore, such a formed material creates a high potential for application in bone-tissue regeneration.

#### 5.2.7. Conjugates of Collagen/HAp/Bioactive Alloys: Preparation and Application

Biometals, due to their good mechanical properties, are the most usually used materials to produce dental and orthopedic implants. However, despite the good mechanical characteristics of biometals, their biological properties, such as osteoconductivity and their abilities in osseointegration, do not meet the requirements of modern multifunctional biomaterials [233]. Because the bone–implant interface is critical for bone ingrowth and early fixation of implants, covering of metallic implants with coatings is one of the most common strategies to enhance surface bioactivity.

Various techniques have been applied for the preparation of HAp/Coll coatings on metal surfaces. Among all methods used for the preparation of HAp/Coll coatings on metals, the most commonly applied are single-step methods, including electrochemically-assisted deposition, as well as methods based on self-assembly of HAp/Coll coating by immersion in a solution containing collagen and various HAp precursors, such as supersaturated calcification solution (SCS) [234,235], SBF [236,237], double-strength simulated body fluid (2SBF) [238], and a solution containing NaH_2_PO_4_, NaCl, CaCl_2_ [239], or Ca(NO_3_)_2_ and NH_4_H_2_PO_4_ [240]. The second subgroup of biomimetic HAp/Coll coatings obtained includes the preparation of composite coatings via a two-step route that involves the HAp layer deposition by self-assembly in simulated body fluid, followed by the incorporation of collagen by soaking in a collagen-containing solution [241,242]. In the two-step route, other techniques of HAp layer preparation, including the hydrothermal method [242], liquid precursor plasma spraying process [243], and aerosol deposition [244], also have been proposed. Preparation of HAp/Coll coatings was also performed with the spin-coating technique [245]. A method based on reverse deposition of components on stainless steel was presented by Tapsir et al. [246]. The authors prepared the surface of steel discs by grafting with a polydopamine, and then collagen fibers were immobilized covalently by immersion in a Coll-containing solution. Finally, disks were biomineralized with HAp in SBF. In Table 2. The methods of HAp/Coll preparation have been summarized.

Benmarouane et al. carried out studies of the bone–implant interface using synchrotron radiation on ID15 at the ESRF in Grenoble, France. They investigated the residual stress and texture of the novel crystals of bone reconstructed with interfacial bone implant titanium (Ti-Al-4V)-coated HAp [247,248].

Osteogenic properties of HAp/Coll coatings were investigated by He et al. [243]. Studies performed by the authors revealed improved mesenchymal stem cell (MSC) adhesion and proliferation on a HAp/Coll coating as compared to an unmodified HAp coating. Moreover, the HAp/Coll coating exhibited higher osteocalcin (OCN) expression, demonstrated enhanced cell differentiation, and had a higher level of osteopontin (OPN) secretion in comparison with uncoated ceramic coating. Comparative studies on osteoblastic MC3T3-E1 cell proliferation and differentiation on Ti substrates, collagen-coated Ti, and HAp/Coll composite coatings revealed no significant differences in the proliferation of cells incubated on Ti and collagen-coated Ti discs. On the other hand, a significant increase in MC3T3-E1 proliferation was recorded for cells seeded on the HAp coating. Similar trend was observed for alkaline phosphatase (ALP) activity of MC3T3-E1 cells seeded on the uncoated Ti substrates and on samples coated with HAp/Coll composites [245]. In vivo investigations on bone formation in the peri-implant area in rabbit tibias implemented with uncoated acid-etched Ti, HAp-coated Ti, and implants coated with HAp/Coll composite were performed by Lee et al. [244]. Histologic images of sliced implant sections revealed greater bone-to-implant contact for HAp/Coll-coated implants (41.45 ± 6.77%) in comparison with acid-etched Ti disc (21.38 ± 6.76%) and HAp-coated (24.18 ± 8.21%) implants. Moreover, greater peri-implant bone formation was observed for composite-coated implants (47.04 ± 17.82%) in comparison with uncoated materials (23.34 ± 13.28%) and HAp-coated Ti (22.85 ± 12.35%). Investigations including histomorphometric analyses and bonding-strength tests of Ti rods with a machined surface coated with HAp and covered with a HAp/Coll composite placed under the periosteum of a calvarium were undertaken by Uezono et al. [236]. The experimental results showed that four weeks after implantation, the uncoated implant and the one covered with the ceramic coating were encapsulated with fibrous tissue, whereas HAp/Coll-coated Ti rods were almost completely surrounded by newly formed bone tissue. Additionally, histomorphometric analyses indicated that the HAp/Coll samples revealed the greatest bone-contact ratio and the greatest bonding strength to bone among all implants. Nanoindentation investigation of the effects of collagen on the mechanical properties of hydroxyapatite coatings revealed the phenomena of strengthening of composite coatings. Incorporation of collagen into the HAp coating resulted in the increase of the hardness and a twofold increase of the Young’s modulus of inorganic–organic coatings in comparison with the HAp coating prepared by soaking in SBF [242].

However, it is worth emphasizing that the success of a coated implant is largely dependent on its stability. The stability of the implant prevents its aseptic loosening and supports osteogenesis around implants, reducing the risk of implant failure [249]. The effect of coating stability on osteointegration performance of coated metallic implants can be examined by numerous techniques, including micro X-ray computed tomography (micro-CT), scanning electron microscopy, and histological examinations by laser scanning confocal fluorescence microscopy [249,250]. However, in order to achieve reliable results of implant-stability investigation, it is also worth considering the use of friction and wear tests of coatings [251], examination of the shear strength of the bone implant interface in an in vivo model [252], and the strength of coating adhesion as well [253].

An investigation on wettability and chemical stability of apatite/collagen composite coatings electrochemically deposited on a NiTi shape memory alloy was performed by Sun et al. [238]. The authors showed that composite-coated NiTi samples were characterized by higher wettability and corrosion resistance in comparison with apatite-coated and uncoated NiTi alloys. Considering the application of these materials in the human body, the presented results suggest that such obtained composite coatings may improve the chemical stability of the metallic implants. In turn, the improved wettability can prevent platelet adhesion and thrombosis, and support osteoblast adhesion and proliferation.

A comparative study on osteointegration of bare Ti rods and composite-coated metal was performed by Uezono et al. [236]. The presented histomorphometrical investigation performed in a rat calvarium showed that four weeks after implantation of rods, the coated implants were completely surrounded by new bone tissue, whereas all the bare samples were encapsulated with fibrous tissue. Moreover, the experiments revealed that among the tested samples, the composite-coated rods exhibited the greatest contact ratio and bonding strength to the bone as well.

However, to the best of our knowledge, there are no reports considering such composite-coated load-bearing implant osteointegration. Moreover, some additional research, including coating adhesion tests and wear resistance trials, are needed to unequivocally state whether hydroxyapatite/collagen-coated metallic materials are suitable for load-bearing applications and long-term usage.

### 5.3. Conjugates of Collagen/HAp/Bioactive Macromolecules

Currently, many scientific works and clinical trials focus on the use of bioactive macromolecules in tissue engineering. Bioactive macromolecules can reduce tissue inflammation and speed up the healing process. The problem with the use of these types of agents is their short biological activity. The biggest challenge today is to improve the delivery and retention of bioactive components. The use of HAp/collagen compositions containing bioactive macromolecules improves the targeted delivery of the active substance [11,254,255,256,257,258].

#### 5.3.1. Conjugates of Collagen/HAp/Proteins: Preparation and Application

**Figure 11:**
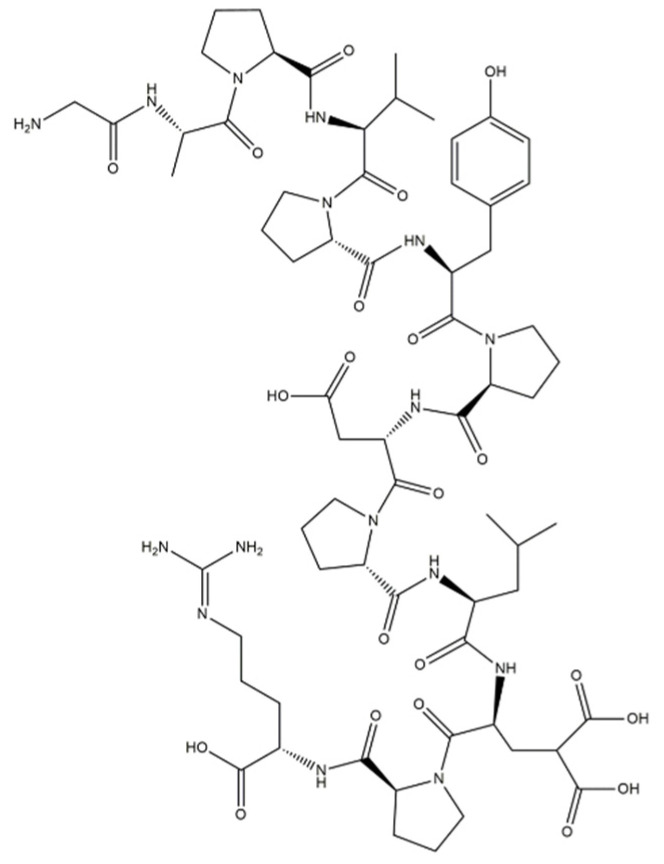
Schematic representation of osteocalcin’s chemical structure.

Some of the most common biomolecules used in regenerative medicine are proteins. These are macromolecular biopolymers, formed from amino acid residues, and combined by a characteristic peptide bond [259]. The most widely described proteins, which are also used clinically, are bone morphogenetic proteins (for example, BMP-2). However, as they are known as growth factors, they are presented more precisely in 5.3.7. Yet another protein of exceptional importance for the regeneration of the skeletal system seems to be osteocalcin, which is an extracellular bone matrix protein. In humans, this small peptide consists of 49 amino acids, and is one of the most abundant, noncollagenous proteins in bone [260]. Osteocalcin (Figure 11), also known as bone γ-carboxyglutamic acid protein, is a factor expressed and secreted solely by osteoblasts [261].

**Figure 12:**
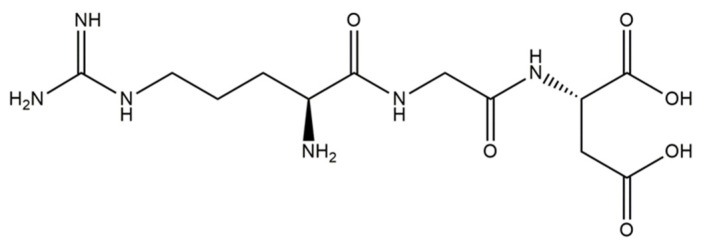
Schematic representation of the RGD peptide’s chemical structure.

In 2002, Knepper-Nicolai et al. [262] described for the first time the tricomponent composite, i.e., calcium/phosphate bone cement with collagen modified with osteocalcin. It was physically analyzed by SEM and AFM, as well as microbiologically tested. The biological analysis was performed with reference to the human osteosarcoma cell line SAOS-2, and the results obtained suggested that osteocalcin may improve initial adhesion of osteoblast-like cells. Similar conclusions also were described in [263], in which the efficiency of the presented protein on wound healing and the bone-remodeling process was investigated. The composites were implanted in the tibial head of an adult Wistar rats. After 56 days, high expression of bone-specific matrix proteins (osteopontin, bone sialoprotein, CD44) were found, and it was shown that osteocalcin activated both osteoclasts and osteoblasts during early bone formation. Another noncollagenous glycoprotein is osteonectin, responsible for calcium ion binding and also perceived as a mineralization-process regulator. It is mainly expressed in tissue undergoing growth, remodeling, or repair [264]. Besides bone, this protein is found in many tissues undergoing morphogenesis, where it is expressed and secreted by different cell types such as cancer cells, endothelial cells, fibroblasts, and VSCM [265]. In the concerned hydroxyapatite/collagen type I systems, it not only affects in vivo bone development, but potentially presents a chance to advance new biomimetic methods for bone-graft applications. However, it has been reported that mineralization of the fibers did not occur for collagen type II [266]. Last, a typically osteogenic protein bound in the HAp/collagen system described in the literature is bone sialoprotein (BSP). It is an acidic phosphoprotein of the ECM that is expressed at high levels in mineralized tissues, and shows ability to bind collagen I and nucleate hydroxyapatite [267]. Moreover, BSP shows the ability to modulate cellular functions such as proliferation, apoptosis, adhesion, migration, and angiogenesis, as well as remodeling of ECM [268]. The nature of binding of BSP to collagen, both recombinant and natural bone extracted BSP, was studied. It was demonstrated that optimal binding between rBSP and collagen was stabilized by hydrophobic interactions. Moreover, this system showed an increase in nucleation potency, thus implying the formation of new mineral layers [269]. An interesting nonosteoprotein, experimentally designed to regenerate the skeletal system, is keratin. It is a fibrous protein with exceptional mechanical properties. Its spiral molecular structure and macromolecular organization affect the high strength and the flexibility of the protein. It is the main component of materials such as hair, fur, wool, nails, hooves, or claws; however, several years ago it was experimentally combined with bioactive ceramics to determine the potential use of such formed material in the regeneration of the skeletal system [270]. Two-component systems using keratin and hydroxyapatite have been used to produce membranes [271], porous scaffolds [272], and laminar and reinforced keratin/HAp scaffolds [273]. However, in 2017, Arslan et al. [274] presented a very cost-effective method of obtaining keratin from human hair (from a local hairdresser after obtaining ethical permission for its use), hydroxyapatite from egg shells, and collagen from the jellyfish *Rhizostoma pulmo*. The designed three-dimensional scaffolds based on obtained substrates were examined biologically with regard to human adipose mesenchymal stem cells. It was reported that the tested cells were successfully kept alive, and after a certain time, they self-differentiated to the osteogenic line without any in vitro inducing agents on the scaffold. Therefore, it is believed that these osteoinductive biocomposite scaffolds with keratin have the potential to be used in bone-tissue engineering. Schneiders et al. [275], using RGD peptide (Figure 12) as a protein building blocker, proposed an equally interesting solution.

The RGD peptide, otherwise known as peptide containing arginine-glycine-aspartic acid (Arg-Gly-Asp), is one of the most commonly used peptides for the functioning of biomaterials [276]. This is due to the fact that it occurs in several proteins, such as collagen I, fibronectin, bone sialoprotein, and osteopontin [277,278]. RGD peptides are mainly responsible for the osseointegration and interaction of cellular integrin receptors with extracellular bone matrix proteins. What is more, it is also an important sequence used in targeted therapy, e.g., in targeted cancer treatments [279]. The authors of the study proved that the addition of RGD to HAp/collagen composite cements appears to enhance bone remodeling at the early stages of bone healing, thus leading to increased bone formation [275].

#### 5.3.2. Conjugates of Collagen/HAp/Phospholipids: Preparation and Application

Phospholipids are a group of organic compounds representing important building blocks of the cell membrane. They are particularly abundant in nerve tissue, liver, and blood; however, their role in bone formation, as well as remodeling, is equally significant. As early as 1986, Raggio et al. [280] described the formation of hydroxyapatite layers under in vivo conditions, induced by the presence of lipids. They based their conclusions on studies on rabbit muscle bags, in which lipids extracted previously from rabbit bones were placed. After three weeks, it was shown that acid phospholipids could cause the deposition of hydroxyapatite minerals in the physiological environment. A few years later, Xu and Yu et al. [281] explored this subject in more detail, describing the mechanism of bone formation at the molecular level. The analysis was carried out on the bovine tibia. It was subjected to demineralization, which revealed small spheres of 145 nm size present in the tested structure. These turned out to be lipids, since they were soluble in organic solvents. Further analysis of the composition of compact bones indicated a high content of nonpolar lipids such as cholesterol ester and triglycerides. The results of the study suggested that a layer of round lipid particles on collagen fibers mediated the deposition of minerals, which induced the formation of apatite layers. Due to the above-mentioned conclusions, as well as the significant role of phospholipids in bone regeneration, studies on such three-component systems with hydroxyapatite and collagen have also been performed. In their work from 2012, Yang et al. [282] decided to create scaffolds using nano-hydroxyapatite, type I collagen, and phosphatidylserine (PS).

**Figure 13:**
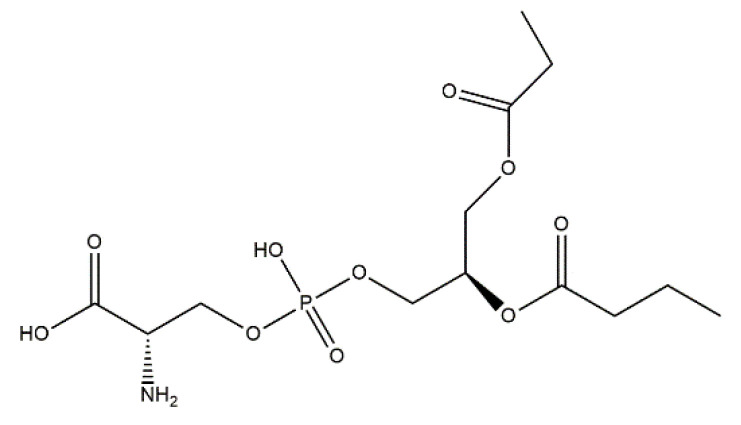
Schematic representation of the chemical structure of phosphatidylserine (PS).

PS is an essential ingredient in all human cells, and is present on the internal leaflet of the cell membrane. It plays a key role in cell cycle signaling, specifically in apoptosis [283]. The chemical structure of PS has been presented in Figure 13.

Exposure of PS on the surface of the vascular endothelium in cancer cells (lung, breast, pancreatic, bladder, skin, brain metastasis, etc.) is caused by oxidative stress [284]. In the aforementioned three-component system, the inorganic part constituted about 65% of the system’s weight. Bone and tissue reactions with the composites were studied in animal tests using rabbit tibias. Research on these biomimetic scaffolds showed an impressive effect. Bone growth was observed periodically by using X-rays of optical micrographs up to 12 weeks. Therefore, HAp/collagen/PS scaffolds can be a suitable substitute in bone-tissue engineering. In 2015, Yang and Fang et al. [285] considered a similar arrangement with a gradient porosity analyzed as a drug carrier. The influence of the scaffold structure on the drug-release kinetics and bioactivity of cells was also determined. Microcapsules of collagen filled with the steroid saponin were placed both in a loose and a dense layer of scaffolds. As a result, the loose layer showed a higher release of the drug compared to the dense layer. Such differences in release kinetics had a clear impact on cell bioactivity. The cell propagated much more in the loose layer than in the dense layer. Thus, the presented system may provide opportunities for tissue regeneration in combination with optimal drug doses at the wound site and lessen potential side-effects at uninjured sites. However, more frequent examples of the discussed three-component composites are those in which the hydroxyapatite phase has been replaced by the other ceramics, e.g., bioglass. It was shown that like the biomimetic bioglass/collagen/PS system [286], scaffolds with separated rat mesenchymal stem cells [287] and a controlled release system modified with steroid saponin [288] fulfilled the basic requirements for forming scaffolds for bone tissue and have the potential to be used in orthopedic and reconstructive surgery.

#### 5.3.3. Conjugates of Collagen/HAp/Pectin: Preparation and Application

**Figure 14:**
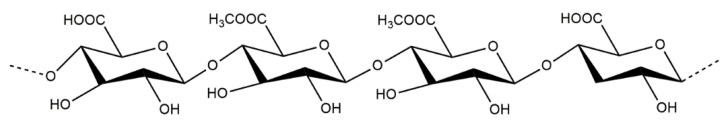
Schematic representation of pectin’s chemical structure.

Structurally and functionally, pectins belong to the group of the most complex polysaccharides occurring in the walls of plant cells. These components participate in plant growth, development, and defense [289]. In the food industry, pectins (Figure 14) are used as gelling agents, and also as a valuable source of roughage. Furthermore, it was reported that these polysaccharides are characterized by various biological activities; i.e., they may decrease the levels of lipids, cholesterol, or glucose, and they exhibit anticancer activity [290,291]. The chemical and structural properties of these polysaccharides allow their interactions with a wide spectrum of molecules, which enables the development of matrices designed for controlled drug delivery [292].

Three-component materials consisting of collagen, hydroxyapatite, and pectin have been described by Wenpo et al. [293]. In their study, the structure of such materials, as well as their mechanical properties, sorption ability, enzymatic degradability, and cytotoxicity, were characterized. SEM and XRD analyses confirmed the presence of an inorganic phase in the form of hydroxyapatite, which was homogenously dispersed in an organic matrix. Results of FT-IR spectroscopy indicated the occurrence of strong interactions between collagen, pectins, and hydroxyapatite. The mechanical properties, enzymatic degradability, and cytotoxicity analysis indicated that the developed composites may be potentially used in bone-tissue engineering.

#### 5.3.4. Conjugates of Collagen/HAp/Natural Polysaccharides: Preparation and Application

**Figure 15:**
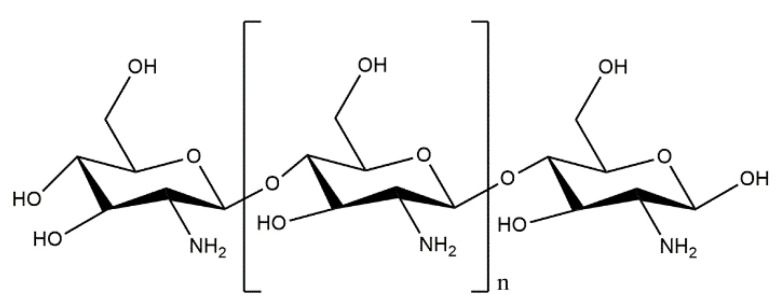
Schematic representation of chitosan’s chemical structure.

Chitin is one of the most frequently occurring natural polysaccharides [294]. It is a linear polymer consisting of 2-acetylamino-2-deoxy-D-glucose units linked by 1,4-β-glycosidic bonds [295]. Chitin is a component of fungal cell walls and the exoskeletons of arthropods. This polysaccharide also occurs in sponges and corals. For industrial or laboratory purposes, it is widely sourced from marine invertebrates such as crabs or shrimps [296]. Due to numerous problems related to chitin processing, this biopolymer is usually applied in its deacetylated derivative, i.e., chitosan [297]. Due to the high biodegradability, nontoxicity and antimicrobial properties, chitosan (Figure 15) is widely used as antimicrobial agent alone or in combination with other natural polymers [298]. It is applied for medical and pharmaceutical purposes, including the use for preparation of matrices for tissue engineering, targeted drug delivery, or healing of hard-to-heal wounds [299,300]. In numerous investigations, chitosan has been mentioned as component of scaffolds for tissue engineering consisting of collagen and hydroxyapatite [301,302,303]. For example, Wang et al. [304] developed a synthetic bone matrix based on collagen and hydroxyapatite. In order to improve the properties of such a matrix, chitosan was added. The artificial matrix was prepared by a phase-separation method. For this purpose, a 4% chitosan solution and the hydroxyapatite powder were added to the collagen solution. Next, the reaction mixture was placed at −30 °C and subjected to a freeze-drying process. In the next step, the cross-linking process using glutaraldehyde was performed. The prepared matrices were rinsed in distilled water and lyophilized. Finally, the obtained materials were tested by transmission and scanning electron microscopies, as well as the X-ray diffraction technique. Furthermore, in vivo studies were conducted to evaluate the ability of the materials received in the repair of bone defects. Results of the research indicated the increased bone metabolism in the place of the implantation of obtained scaffold, which showed the great application potential of such materials.

**Figure 16:**
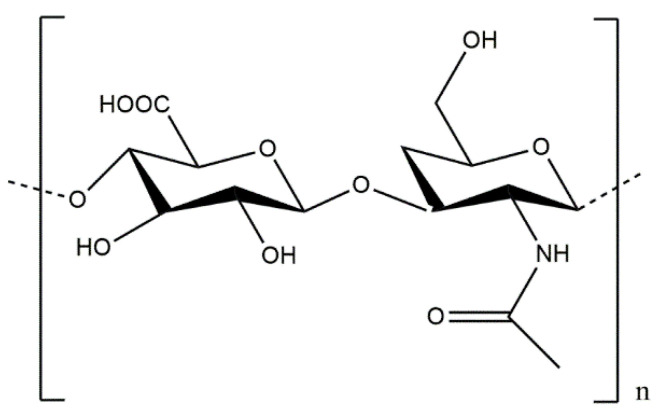
Schematic representation of the chemical structure of glycosaminoglycans.

Kaczmarek et al. [305] focused on the preparation of scaffolds based on chitosan, collagen, glycosaminoglycans (Figure 16), and hydroxyapatite nanoparticles. The obtained composites were characterized in view of the mechanical and physicochemical properties. Furthermore, their sorption properties and biocompatibility also were examined. It was stated that the inorganic additive improved the mechanical properties of the tested composites and increased their stability in an aqueous environment. SEM analysis confirmed the preparation of composites with a porous structure, with a pore size of approximately 250 μm. Results of the biological tests indicated the increased viability of human osteosarcoma SaOS-2 cell lines for materials containing hydroxyapatite. Next, Munhoz et al. [306] performed investigations on the evaluation of bone formation in the place of bone defects experimentally induced in rat skulls. A composite consisting of bovine tendon collagen, chitosan, and hydroxyapatite was used as a material for filling the bone defect. Based on the macroscopic and radiographic investigations, the tested materials were defined as biocompatible. Furthermore, the conducted studies showed that the essential factor determining the osteogenesis process was the concentration of hydroxyapatite. However, the period of the conducted experiment (3–8 weeks) was not enough to achieve full bone regeneration. This probably resulted from the fact that subcutaneous bones, such as the skull, exhibit relatively low osteogenic properties.

Teng et al. [307] proposed a collagen/chitosan/hydroxyapatite combination as composite microspheres. In order to obtain such microspheres, first a 2% collagen/chitosan solution in acetic acid was prepared. Next, an adequate amount of Ca(NO_3_)_2_ × 4H_2_O was introduced into the previously prepared solution and was subsequently emulsified in soybean oil containing surfactant. After the emulsification process, cross-linking with 25% glutaraldehyde solution was performed. Next, Na_2_HPO_4_/NaOH were added dropwise to the emulsion, and the entire solution was intensively mixed. The final step involved a further addition of glutaraldehyde, followed by centrifugation, rinsing, and drying of the microspheres obtained. FT-IR spectroscopy and X-ray diffraction showed that the inorganic phase in obtained the microspheres was crystalline hydroxyapatite containing carbonate ions. However, based on the results of the SEM analysis, it was proved that obtained microspheres were characterized by a narrow particle size distribution, i.e., 5–10 nm. The main objective of the research of Rahman et al. [308] was to develop scaffolds that could be used for regeneration of damaged bone. For this purpose, collagen type I, chitosan derived from shrimps, and bovine hydroxyapatite were used as raw materials. In the first step, physicochemical characteristics of these raw materials was performed. Their biocompatibility, cytotoxicity, and degradability were evaluated. Next, a composite scaffold was obtained by a thermal phase-separation technique. Such a scaffold was crosslinked by various methods, including the use of glutaraldehyde, 2-hydroxyethyl methacrylate, hydrothermal treatment, or IR radiation. As a result of the performed analyses, it was stated that all the developed scaffolds were characterized by a porous structure. Composites crosslinked using IR radiation or glutaraldehyde exhibited better hydrophilicity and biodegradability than the other ones. Furthermore, in the case of all materials, biocompatibility and noncytotoxicity were proved. In the next work, matrices based on collagen and chitosan containing additionally chondroitin sulfate were developed. Results of the XRD technique confirmed the preparation of three-dimensional structure of chitosan/collagen/chondroitin sulfate matrix with homogeneously distributed hydroxyapatite crystals. Next, osteoblastic activity was determined by means of osteoblasts and osteoclasts. It was stated that the matrix morphology resembled a structure of a spongy bone, and improved the bone regeneration while limiting its resorption, and as a result, constituted an interesting biomaterial for bone-tissue engineering [309]. Synthesis of three-component scaffolds based on collagen, hydroxyapatite, and chitosan also was a main research subject of Pallela et al. [310] A scaffold containing chitosan, hydroxyapatite derived from *Thunnus obesus* bone, and marine sponge collagen (*Ircina fusca*) was obtained by the lyophilization method. The structural composition of such formed material was determined by FT-IR spectroscopy. A thermal analysis performed allowed the authors to report an increase in the thermal stability of the scaffold containing hydroxyapatite and collagen. Furthermore, a higher intensity of cell proliferation in the composite scaffolds compared to the scaffolds based only on chitosan was observed.

#### 5.3.5. Conjugates of Collagen/HAp/Synthetic Polymers: Preparation and Application

**Figure 17:**
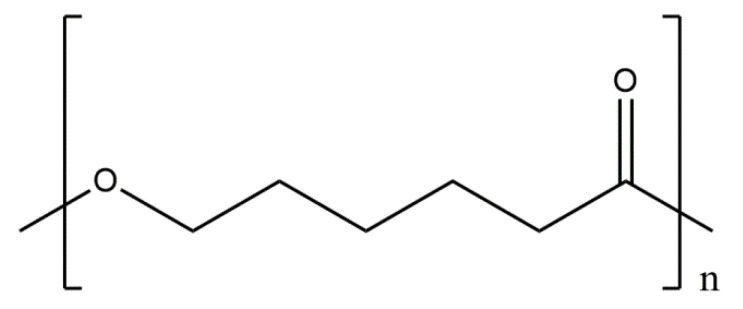
Schematic representation of polycaprolactone’s chemical structure.

**Figure 18:**
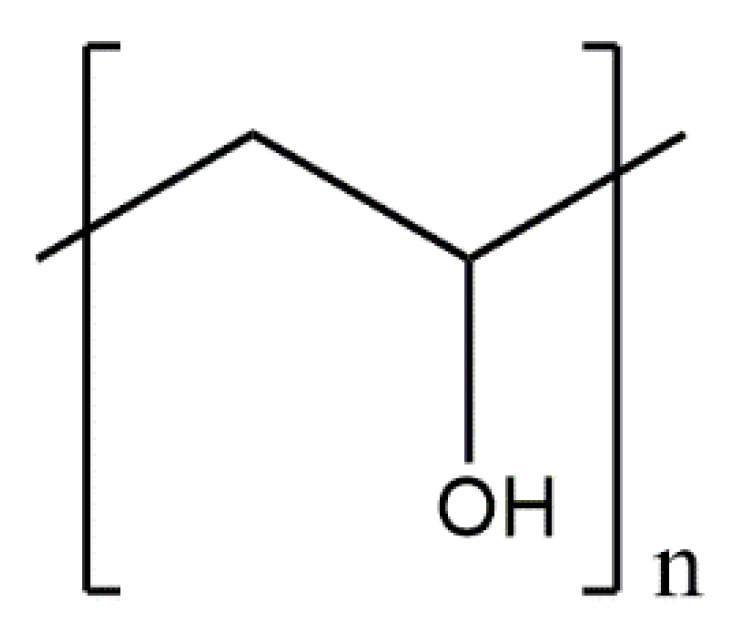
Schematic representation of the chemical structure of poli(vinyl alcohol) (PVA).

**Figure 19:**
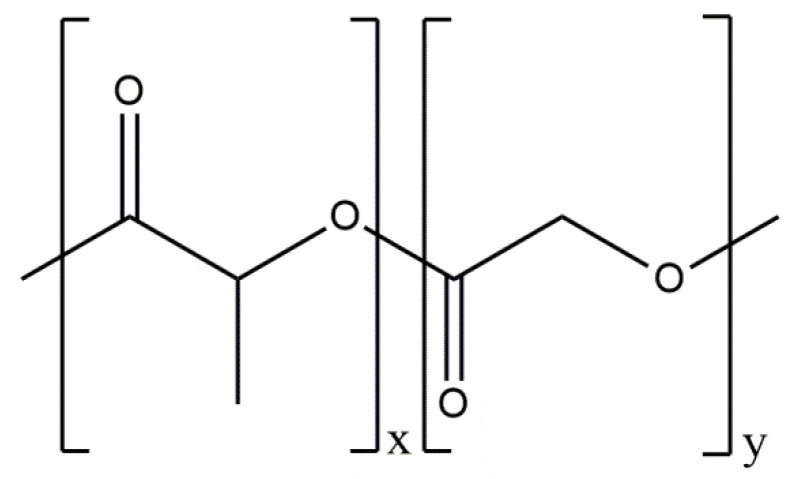
Schematic representation of the chemical structure of poly(lactide-co-glycolide).

Conjugates of collagen/HAp/synthetic polymers also seem to be an interesting group of materials with great application potential. Among synthetic polymers that may be applied in the synthesis of the conjugates are polycaprolactone, poli(vinyl alcohol) (PVA), or poly(lactide-co-glycolide) The chemical structures of these compounds are presented schematically in Figure 17, Figure 18 and Figure 19.

Among the combinations of collagen/hydroxyapatite/polymer, the polymer components are mainly polycaprolactone (Figure 17) [311], poly(vinyl alcohol) (Figure 18) [312] and poly(lactide-co-glycolide) (Figure 19) copolymer [313]. For example, the research subject of Prosecka et al. [314] was to develop a three-dimensional scaffold based on type I collagen and hydroxyapatite enhanced with polycaprolactone nanofibers, autologous mesenchymal stem cells (MSC) in the osteogenic medium, and with a solution containing thrombocytes (TRC). Three types of collagen/hydroxyapatite/polycaprolactone (coll/HAp/PCL) scaffolds were developed: enhanced with MSC, enhanced with RS, and enhanced with both MSC and TRS. In order to assess the impact of the scaffolds on the bone-regeneration process, in vivo investigations were performed. After 12 weeks of implantation, microscopic and histological analyses of the regenerated tissue were conducted. It was reported that the greatest volume and homogeneous distribution of newly formed tissue occurred in the case of coll/HAp/PCL scaffolds enhanced both with MSC and TRS. Furthermore, during the studies on the mechanical strength of the scaffolds, it was stated that the use of PCL fibers resulted in the improvement of the biomechanical properties of the scaffold. On the other hand, Phipps et al. [315] carried out studies on the preparation of a bone-imitating nanofiber scaffold. Scaffolds obtained from the mixture of type I collagen, polycaprolactone, and hydroxyapatite with a dry matter ratio of PCL/coll/HAp equal to 50/30/20 were analyzed. Properties such as biocompatibility and cytotoxicity were determined in comparison to 100% polycaprolactone and 100% collagen, and to the two-component scaffold containing 80% PCL/20% HAp, respectively. Based on the scanning electron microscopy, fluorescent cell imaging and MTS tests, it was stated that mesenchymal stem cells adhered to all obtained scaffolds except the one based on collagen. In the case of studies performed for bone-imitating three-component scaffolds, a more intense proliferation of cells was observed. Three-component scaffolds adsorbed the adhesion proteins and the fibronectin that may contribute to the positive reaction of the cells toward the tested materials significantly faster. Next, Bhuiyan et al. [316] proposed a biomaterial based on collagen, nanohydroxyapatite (nHAp), and poly(lactide-co-glycolide) (PLGA) copolymer. Mechanical properties, as well as the osteogenic potential of this composite, were characterized in order to evaluate the possibility of the use of such material in bone-tissue regeneration. It was reported that human mesenchymal stem cells (hMSC) remained viable on coll/nHAp/PLGA composites. What is particularly important was that it was observed that after seven days of the culture, the population of tested cells increased several times. Over the five days of culture, hMSC deposited the matrix consistently with osteogenic differentiation and the bone formation. Li et al. [317] also carried out studies on the combination of hydroxyapatite, collagen, and a synthetic polymer, i.e., PLGA. The main objective of their work was to develop a new methodology of homogeneous introduction of hydroxyapatite and collagen into the polymer scaffold. For this purpose, the suspension of hydroxyapatite and collagen was mixed with paraffin microspheres, and such prepared mixture was used to obtain a HAp/coll composite. Next, the polymer solution was poured drop by drop into the HAp/coll/paraffin scaffold to completely fill the area between the paraffin microspheres. The last step involved the removal of the paraffin. For this purpose, the composite was immersed in cyclohexane for an adequate period of time (6 h) and subsequently lyophilized. Based on the conducted analyses, i.e., X-ray diffraction and scanning electron microscopy (SEM), it was reported that the proposed method was effective for the preparation of polymer scaffolds containing hydroxyapatite and collagen.

The next example of the combination of hydroxyapatite and collagen also was presented by Degirmenbasi et al. [318]. Hydroxyapatite nanoparticles (n-HAp) with a size of 10–50 nm were obtained separately or via an in situ method in a collagen/poli(vinyl alcohol) (PVA) system.

Ca(NO_3_)_2_·4H_2_O and Na_3_PO_4_ were used as hydroxyapatite precursors. It was stated that the obtained biocomposites HAp/coll/PVA were characterized by a relatively large elasticity that could be additionally increased by cryogenic treatment. Furthermore, SEM investigations confirmed the preparation of materials with a porous structure. Ficai et al. [319] also carried out studies on the development of materials based on PVA. In their work, two types of hybrid materials were presented, i.e., collagen/poly(vinyl alcohol) (coll/PVA) and collagen/poly(vinyl alcohol)/hydroxyapatite (coll/PVA/HAp). During their synthesis, two methods of drying were employed, i.e., controlled air drying at 30 °C and freeze-drying. The prepared composites were investigated using an X-ray diffraction (XRD) technique, FT-IR spectroscopy, and scanning electron microscopy (SEM). Furthermore, the measurements of density, porosity, and xylene absorption also were performed. Based on the SEM images obtained and the porosity analysis, it was reported that the use of PVA resulted in the preparation of materials with a stratified morphology. Importantly, the method of drying also influenced the structure of the material obtained. It was stated that the freeze-dried composites exhibited a higher porosity, while the controlled air drying led to the preparation of materials with a compact structure.

#### 5.3.6. Conjugates of Collagen/HAp/Flavonoids: Preparation and Application

**Figure 20:**
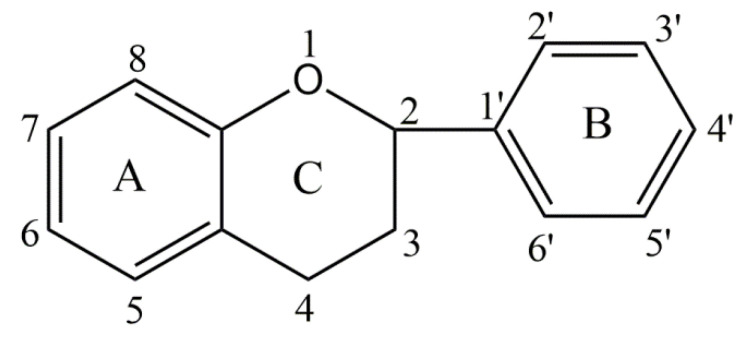
Basic skeleton of C6-C3-C6 compounds.

Flavonoids are plant secondary metabolites belonging to a large group of polyphenolic compounds. Flavonoids’ carbon framework is characterized by a C6-C3-C6 skeleton exhibiting the structure of a chromane or that of a chromene formed by a fused benzene ring (A), the 3,4-dihydro-2H-pyran, or the pyran (ring C) and a phenyl group (ring B) substituted on ring C (Figure 20) [320,321].

Due to structural differences, such as oxidation and hydroxylation levels, as well as substitution of the C ring, flavonoids can be divided into a few major classes, including flavanols, flavanones, flavones, flavonols, anthocyanidines, isoflavonoids (compounds with a 3-phenyl-4H-chromen- 4-one), and neoflavonoids (compounds with a 4-phenyl-2H-chromen-2-one) [322,323,324].

Beneficial health-promoting properties of this group of compounds have been well known for years. Preclinical studies, as well as short-term randomized controlled tests, have demonstrated that flavonoids reduce the risk of cancer [325,326,327,328], exhibit beneficial health effects on cardiovascular and metabolic health [329,330,331], and play an important role in inflammation [332,333], which is involved in several stages of diseases such as asthma [334,335,336], diabetes [337,338,339], or neurodegenerative diseases [340,341,342,343]. Moreover, flavonoids display antiviral properties [344,345,346,347]. Additionally, numerous studies proved that some flavonoid compounds promote osteoblast differentiation and bone formation [348,349,350,351,352,353,354,355], while others prevent resorption of bone [356,357,358,359,360] or ameliorate arthritis [361,362,363,364,365,366,367].

Due to the well-proven bone-regeneration-supportive properties of flavonoids, their application as active constituents of biomaterials including hydroxyapatite and collagen is understandable. Functionalization of HAp with various flavonoids leads to enhanced human osteoblastlike MG63 proliferation and differentiation [368], osteogenic differentiation of osteoblast-like cells and MSCs [369], downregulation of inflammatory cytokine expression from macrophage-like cells [370], inhibition of biofilm formation related to periodontitis [371], and hindering of cell damage related to oxidative stress [372]. In turn, studies performed with animal models clearly indicated that flavonoid collagen grafts were more effective in regeneration of bone than a collagen matrix alone [373], have the potential to accelerate the formation of bone tissue in early healing stages [374], and exhibit anti-inflammatory activity by downregulating inflammatory-related genes [375]. Flavonoid-loaded collagen materials are also promising agents in the regeneration of cartilage [376] and nerves [377], as well as in dermal wound dressings [378]. Nature-derived active agents are especially attractive for the sake of medicinal benefits, as well as the reduction of scaffold costs.

**Figure 21:**
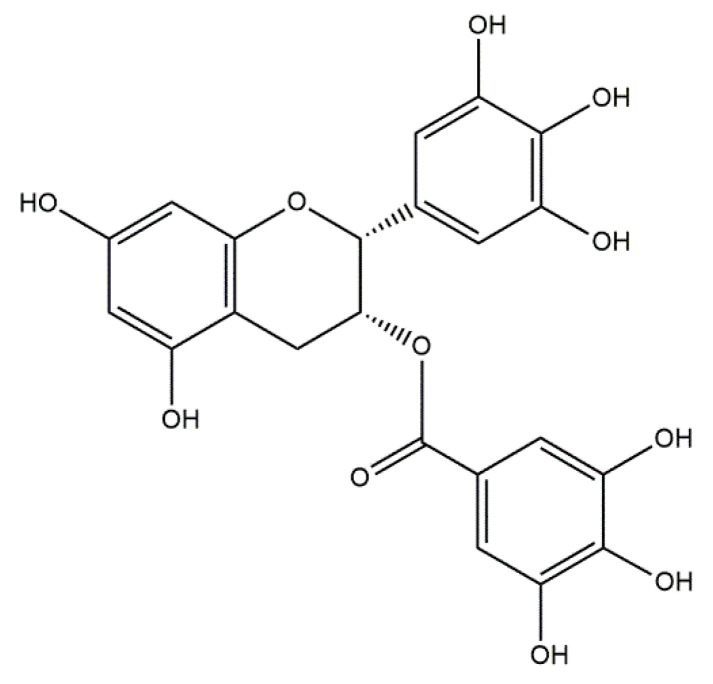
Schematic representation of the chemical structure of epigallocatechin gallate (EGCG).

In recent years, considering the multidirectional beneficial properties of HAp, collagen, and flavonoids, the attention of scientists has been drawn to ternary composite materials. Investigations on duck’s feet collagen/hydroxyapatite sponges containing epigallocatechin gallate (EGCG) (Figure 21) (an ester of epigallocatechin and gallic acid) were undertaken by Kook et al. [379].

The sponges were obtained by utilizing the freeze-drying method. The as-prepared materials were characterized by porosity in the range of 47.35 ± 5.53 to 60.42 ± 2.94% and compressive strength dependent on EGCG concentration. It was found that the addition of EGCG resulted in an increase in the compressive strength. The SEM analysis revealed the interconnected pore structure of composites, which is critical for transporting essential nutrients and is known to improve bone regeneration in vivo [380]. The experiments also included investigations of the proliferation of bone marrow stromal stem cells (BMSCs) on the sponges using MTT (3-4-2,5-diphenyl tetrazolium bromide) and alkaline phosphatase activity (ALP). For the sake of detection of the expressions of bone-specific genes, the ability of cells to osteogenic differentiation on sponges was studied with reverse-transcription polymerase chain reaction (RT-PCR). All the performed in vitro experiments confirmed the suitability of materials containing EGCG for further biological evaluation due to the favorable effects of EGCG on osteogenic differentiation of BMSCs. The prepared materials were also tested in vivo. Histological analysis of sponges implanted under the subcutaneous region of mice revealed the formation of a thicker bone matrix, higher osteoblast infiltration, and calcium deposition for the EGCG-containing sponges as compared to collagen/hydroxyapatite materials. The presented results indicated that the obtained composite sponges were promising materials for stimulating bone regeneration. However, it is worth emphasizing that the experiment showed the flavonoid-dose-dependent biological activity of such materials, thus determining the appropriate EGCG concentration is of crucial importance [379].

**Figure 22:**
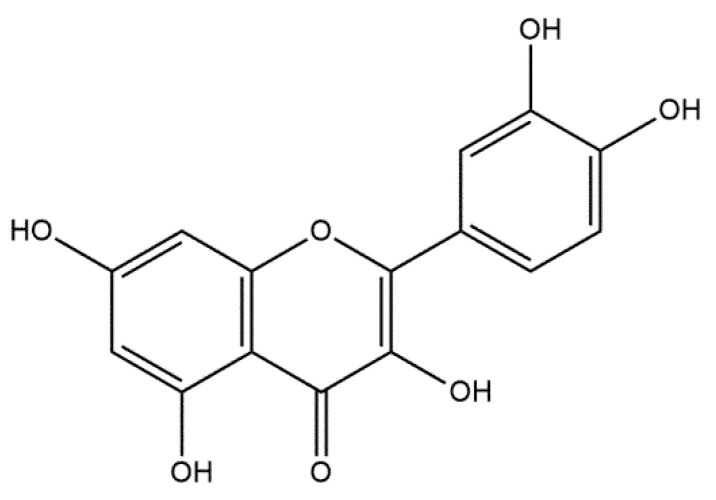
Schematic representation of quercetin’s chemical structure.

Similar research was performed by Song et al. [381]. Their paper describes composite sponges prepared by lyophilization and composed of collagen isolated from duck’s feet, hydroxyapatite, and quercetin (Qt) (Figure 22), a compound belonging to the flavonol subgroup. The SEM analysis of the morphology of such obtained materials revealed their uniform 3D structure with well-oriented pores. Moreover, SEM imaging confirmed adhesion of BMSCs to the surface and pores of sponges.

The effect of flavonoid addition on the toxicity of materials was evaluated with an MTT assay, which revealed gradually increasing proliferation of cells seeded on the sponges over time. Osteogenic differentiation of BMSCs on the composites was evaluated by RT-PCR by examining the expressions of bone-specific genes such as OCN, considered as a middle and late marker of osteogenic differentiation, and RUNX-2 and COL1, which are recognized as early markers. The obtained results showed that a 25 µL addition of Qt resulted in stimulation of osteogenic differentiation at various stages. In vivo evaluation of the osteoconductive ability of materials was performed in the rat model implementing sponges in an animal calvarial bone defect. Studies revealed that sponges containing 25 μM of Qt promoted bone regeneration to a greater extent in comparison with the material composed only of hydroxyapatite and collagen. The presented outcomes indicated that Qt-incorporated HAp/Coll sponges may be applied as bone-regeneration-supporting materials and as scaffolds for tissue engineering.

**Figure 23:**
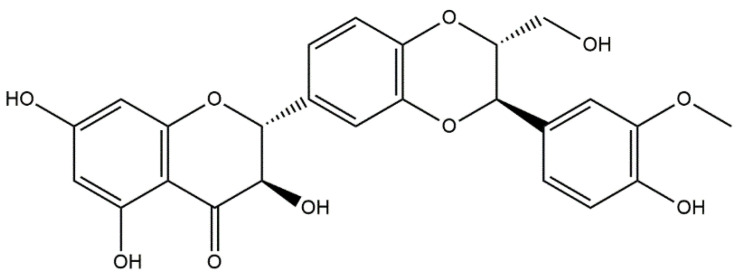
Schematic representation of silymarin’s chemical structure.

Another flavonoid tested by the same research group as an active ingredient of HAp/Coll sponges was silymarin (Sm) (Figure 23) [382]. The Sm-containing HAp/Coll sponges were prepared by freeze-drying. In contrast to the materials discussed above, the structure of Sm-containing composites was characterized by the irregular shape of pores. The porosities of materials were 77.5% and 83.5%, for HAp/coll and 100 μM Sm/HAp/Coll, respectively. BMSCs seeded on the materials revealed their spindlelike shape and secreted extracellular matrix to develop a connection network. Both the density of the network and the amount of the secreted matrix increased with the Sm concentration. MTT assay results confirmed that the rate of proliferation increased with the increase in the concentration of Sm. The results of the measurement of ALP activity and expression of markers (OCN, COL1, and RUNX-2) showed a similar tendency. The obtained materials were tested in vivo with the use of the animal models. Eight weeks after implantation, it was noticed that defects were nearly filled with newly formed tissue organized in osteoids, proving the influence of Sm on the bone-regeneration process. Interestingly, in contrast to the previously discussed research, in this case no negative effects of a higher dose of the compound on the bone-regeneration process were demonstrated.

**Figure 24:**
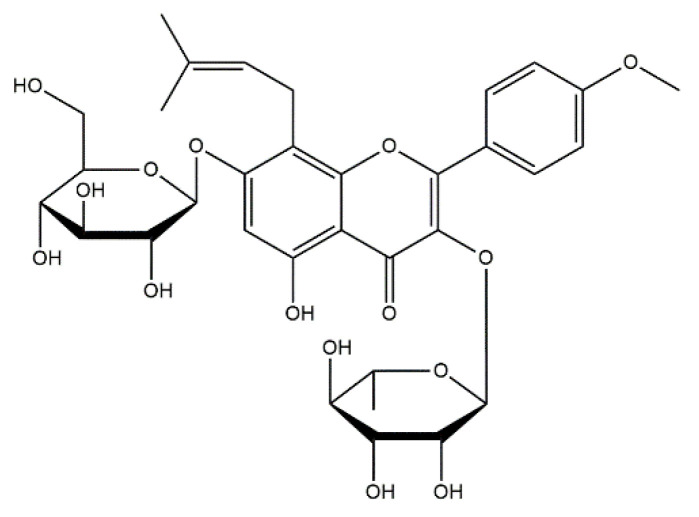
Schematic representation of the chemical structure of icariin (Ic).

Interesting core-shell multicomponent materials mimicking the structure of bone were proposed by Zhao et al. [383]. The authors obtained material composed of HAp/Coll/polycaprolactone (PCL) electrospun shell and a freeze-dried core consisted of a collagen with icariin (Ic) (Figure 24) loaded chitosan microspheres. The core-shell materials were cross-linked by genipin. The electrospun shell displayed a three-dimensional network with a fiber diameter in the range of 300 nm to 1000 nm. Investigations on rat marrow mesenchymal stem cell (rMSC) attachment to the core and shell of scaffolds revealed a well-attached cell layer on the surface of the materials, which was also confirmed by fluorescence staining. Bone-regeneration processes were studied by implementing materials with Ic-loaded microspheres (CHPI) and composites containing Ic-unloaded microspheres (CHP) into the defect in tibial plateau created in white rabbits. After 12 weeks of implantation of CHP, bone defects were not completely filled, whereas CPHI defects were filled with a bone mass at eight weeks. Moreover, the formation of new bone around and in the center of the implant was recorded for the CPHI group in the fourth week, while this effect was not recorded for the Ic-free materials. The obtained results indicated that Ic is an effective agent for inducing bone regeneration, and Ic-loaded materials have a great potential for application in tissue engineering.

#### 5.3.7. Conjugates of Collagen/HAp/Growth Factors: Preparation and Application

Growth factors such as cytokines play an important role in the behavior of cells. They affect cell migration, adhesion, proliferation, and differentiation. Bone healing is a dynamic and multistage process with the following phases: inflammation, cartilage formation, cartilage resorption, primary bone formation, secondary bone formation, and bone remodeling [11,384,385,386,387] (Table 3).

Many cytokines are involved in the healing process. The most important function of bone morphogenetic proteins is to support, in conjunction with other growth factors and cytokines, the formation of cartilage and new bone. In the first stage of the development of growth-factor delivery systems, an attempt was made to recreate the organic or mineral part of bone. In the beginning, collagen sponges and hydroxyapatite were used because of their similarity to natural bone. Due to the brittleness of hydroxyapatite ceramics and insufficient mechanical properties of collagen, in particular low flexural and compressive strength, Hap/collagen compositions have been used. Due to their properties, composite materials can be used as carriers for the delivery of growth factors in bone tissue engineering. Another type of Collagen/HAp/GF conjugates are collagen hydrogels. GF and HAp are incorporated directly into the collagen matrix. Kanematsu et al. [388] developed a collagen-based hydrogel formed in solutions of basic fibroblast growth factor (bFGF), hepatocyte growth factor (HGF), platelet growth factor (PDGF-BB), VEGF, insulin-like growth factor-1 (IGF-1), and binding heparin epidermal growth factor-like growth factor (HB-EGF). VEGF, HB-EGF, and IGF-1 showed a characteristic burst-release profile and a significant loss of loaded GF. However, HGF, bFGF, and PDGF-BB showed somewhat sustained release profiles that were parallel to the biodegradation profile of the collagen matrix. Depending on the pH, the collagen–GF interaction can be enhanced. Based on studies with recombinant human bone morphogenic protein 2 (rhBMP-2), it was found that the binding of rhBMP-2 to collagen could be increased not only by increasing the pH, but also by adding NaCl to the carrier solution. Proton ions have been found to facilitate the self-association of rhBMP-2 to form a layer on collagen. Thus, rhBMP-2 could be loaded more efficiently into collagen hydrogels, yielding improved osteoinductive materials [389].

Geiger et al. [390] confirmed that collagen-containing compositions have been used frequently as growth-factor delivery systems in recent years. Another significant problem when using growth factors is their short shelf-life after application. In the case of bone morphogenetic protein (BMP), especially BMP-2 and BMP-7, it takes about 10 min [391]. There has also been information on serious side effects caused by the use of high concentrations of BMP-2 in commercial biomaterials. Side effects of BMP-2 include postoperative inflammation and related side effects, ectopic bone formation, osteoclast-mediated bone resorption, and inappropriate adipogenesis. Several studies have confirmed the occurrence of adverse events associated with the clinical use of BMP-2, including life-threatening cervical spine edema. The Food and Drug Administration has issued a warning about the potential life-threatening complications of BMP-2 [392,393,394,395,396].

## 6. Conclusions and Perspectives

With the rapid advances in tissue engineering and materials science, various compositions that play a very important role in biomedical applications are being developed. For many reasons, materials designed from collagen and hydroxyapatite ceramics play a meaningful role in the regeneration of bone tissues. Many studies are currently being performed on the incorporation of active substances into collagen/HAp composites. In this review, we summarized collagen/HAp compositions modified with substances such as drugs, metals, nanoparticles, and bioactive macromolecules. HAp/Col compositions are distinguished by high biocompatibility, bioactivity, osteoconductivity, and bioresorbability. Collagen/Hap biomaterials play an important role in drug delivery and slow and sustained release. Various drugs, such as antibiotics, can be added to these types of biomaterials to inhibit the growth of bacterial strains that have caused severe wound infections, and to support tissue regeneration. The modification of conjugates of collagen/HAp with metals and nanoparticles plays a significant role in biomedical engineering. Metals such as cisplatin and magnetic nanoparticles may play an important role in anticancer therapy. On the other hand, the addition of nanotubes and graphene improves the mechanical properties, flexibility, and biocompatibility of collagen/HAp compositions. Various bioactive macromolecules have been added to collagen/Hap compositions to reduce tissue inflammation and accelerate the healing process. An important issue is the optimization and improvement of the compatibility between all the components of such formed compositions. It is important to enhance the synergistic effect of all components of the composition in order to achieve new biofunctional materials that accelerate bone-tissue regeneration. An important issue is the durability and biological activity of additives in the collagen/HAp matrix. To achieve this, it is necessary to understand the interactions between the various substances. In order to develop these biomaterials with application potential for regenerative medicine, further research is needed on collagen/HAp compositions modified with active substances.

## Figures and Tables

**Table 1:** Classification of collagens according to their molecular structure.

Type	Molecular Composition	Tissue Distribution	Genes (Genomic Localization)	Supramolecular Structure and Organization
I	[a1(I)]2a2(I)	bone, dermis, tendon, ligaments, cornea	COL1A1 (17q21.31–q22) COL1A2 (7q22.1)	Fibril-forming collagens
II	[a1(II)]3	cartilage, vitreous body, nucleus pulposus	COL2A1 (12q13.11–q13.2)	
III	[a1(III)]3	skin, vessel wall, reticular fibers of most tissues (lungs, liver, spleen, etc.)	COL3A1 (2q31)	
V	a1(V),a2(V),a3(V)	lung, cornea, bone, fetal membranes; together with type I collagen	COL5A1 (9q34.2–q34.3) COL5A2 (2q31) COL5A3 (19p13.2)	
XI	a1(XI)a2(XI)a3(XI)	cartilage, vitreous body	COL11A1 (1p21) COL11A2 (6p21.3) COL11A3 = COL2A1	
IV	[a1(IV)]2a2(IV); a1–a6	basement membranes	COL4A1 (13q34) COL4A2 (13q34) COL4A3 (2q36–q37) COL4A4 (2q36–q37) COL4A5 (Xq22.3) COL4A6 (Xp22.3)	Basement-membrane collagens
VI	a1(VI),a2(VI),a3(VI)	widespread: dermis, cartilage, placenta, lungs, vessel wall, intervertebral disc	COL6A1 (21q22.3) COL6A2 (21q22.3) COL6A3 (2q37)	Microfibrillar collagen
VII	[a1(VII)]3	skin, dermal–epidermal junctions, oral mucosa, cervix	COL7A1 (3p21.3)	Anchoring fibrils
VIII	[a1(VIII)]2a2(VIII)	endothelial cells, Descemet’s membrane	COL8A1 (3q12–q13.1) COL8A2 (1p34.3–p32.3)	Hexagonal network-forming collagens
X	[a3(X)]3	hypertrophic cartilage	COL10A1 (6q21–q22.3)	
IX	a1(IX)a2(IX)a3(IX)	cartilage, vitreous humor, cornea	COL9A1 (6q13) COL9A2 (1p33–p32.2)	FACIT collagens
XII	[a1(XII)]3	perichondrium, ligaments, tendon	COL12A1 (6q12–q13)	
XIV	[a1(XIV)]3	dermis, tendon, vessel wall, placenta, lungs, liver	COL9A1 (8q23)	
XIX	[a1(XIX)]3	human rhabdomyosarcoma	COL19A1 (6q12–q14)	
XX	[a1(XX)]3	corneal epithelium, embryonic skin, sternal cartilage, tendon	COL21A1 (6p12.3–11.2)	
XXI	[a1(XXI)]3	blood vessel wall	COL21A1 (6p12.3–11.2)	
XIII	[a1(XIII)]3	epidermis, hair follicle, endomysium, intestine, chondrocytes, lungs, liver	COL13A1 (10q22)	Transmembrane collagens
XVII	[a1(XVII)]3	dermal–epidermal junctions	COL17A1 (10q24.3)	Multiplexins
XV	[a1(XV)]3	fibroblasts, smooth muscle cells, kidney, pancreas	COL15A1 (9q21–q22)	
XVI	[a1(XVI)]3	fibroblasts, amnion, keratinocytes	COL16A1 (1p34)	
XVIII	[a1(XVIII)]3	lungs, liver	COL18A1 (21q22.3)	

**Table 2:** Summary of the methods used for HAp/Coll preparation.

Substrate	Deposition of HAp	Deposition of Collagen	HAp Precursors	Collagen Type	Ref.
Ti plate	Single-step electrochemically-assisted deposition		Ca(NO_3_)_2_, NH_4_H_2_PO_4_	Type I extracted from equine Achilles tendon	[240]
Ti-6Al-4V of medical grade	(1) Self-assembly in SBF; hydrothermal method	(2) Iimmersion in collagen solution	SBF; CaHPO_4_×2H_2_O, Ca(OH)_2_	Type I extracted from porcine skin	[242]
Plate-shape dental implants (CP Ti)	Single-step self-assembly		CaCl_2_·2H_2_O, NaH_2_PO_4_×H_2_O, NaHCO_3_ (supersaturated calcification solution)	Type I from bovine Achilles tendon	[234]
NiTi shape memory alloy disks	Single-step electrochemically assisted deposition		Double-strength simulated body fluid (2SBF)	Type I	[238]
Ti-6Al-4V disks	(1) Liquid precursor plasma-spraying process	(2) Immersion in collagen solution	Liquid HAp precursor prepared through a wet-chemical route	Type I derived from bovine skin	[243]
CP Ti discs	Spin-coating of the HAp/Coll sols		Ca(OH)_2_, H_3_PO_4_	Type I derived from calf skin	[245]
Ti disc or implant	(1) Aerosol deposition	(2) Immersion in collagen solution	Commercial HAp powder	Type I from calf skin	[244]
CP Ti screw-shaped dental implants	Single-step self-assembly		CaCl_2_·2H_2_O, NaH_2_PO_4_×H_2_O, NaHCO_3_ (supersaturated calcification solution)	Type I from bovine Achilles tendon	[235]
CP Ti rod	Dipping rod several times into the suspension		Ca(OH)_2_, H_3_PO_4_	Not specified	[236]
Ti-6Al-4V discs	Single-step self-assembly		SBF	Type I extracted from rat tails	[237]
Sintered porous Ti substrate	(1) Immersion in SBF	(2) Immersion in collagen solution	SBF	Humanlike collagen	[241]
Ti plate	Single-step electrochemically assisted deposition		CaCl_2_, NaH_2_PO_4_, NaCl	Type I	[239]
Polydopamine grafted stainless steel (SS316L)	(2) Immersion in SBF	(1) Immersion in collagen solution	SBF	Type I	[246]

**Table 3:** Bone-healing stages involving different biological processes and cytokines [11,384,385,386,387].

Bone Healing Stages	Cytokines	Biological Process Steps
Inflammation	IL-1, IL-6 TGF-β BMP-2 PDGF GDF-8 RANKL and M-CSF OPG	Hematoma Inflammation MSCs recruitment
Cartilage formation	TGF-β2 TGF-β3 BMP-5, BMP-6 VEGFs Angiopoietin-1, Angiopoietin-2	Chondrogenesis Endochondral ossification Cell proliferation Intramembranous ossification Vascular ingrowth New angiogenesis
Cartilage resorption and primary bone formation	VEGF Angiopoietin BMP-3, BMP-4, BMP-7, BMP-8 RANKL & M-CSF	Chondrocyte apoptosis Matrix proteolysis Cartilage resorption Active osteogenesis Bone cell recruitment Woven bone formation Neo-angiogenesis
Secondary bone formation and remodeling	IL-1, IL-6 RANKL and M-CSF	Bone remodeling Osteoblast activity Marrow establishment

## Data Availability

The data that support the findings of this study are contained within the article.
